# Spatial organization and stochastic fluctuations of immune cells impact clinical responsiveness to immunotherapy in melanoma patients

**DOI:** 10.1093/pnasnexus/pgae539

**Published:** 2024-11-26

**Authors:** Giuseppe Giuliani, William Stewart, Zihai Li, Ciriyam Jayaprakash, Jayajit Das

**Affiliations:** Department of Physics, The Ohio State University, Columbus, OH 43210, USA; Steve and Cindy Rasmussen Institute for Genomic Medicine, Nationwide Children's Hospital, Columbus, OH 43205, USA; GIG Statistical Consulting, LLC, Columbus, OH 43205, USA; Pelotonia Institute for Immuno-Oncology, The Ohio State University, Columbus, OH 43210, USA; Department of Internal Medicine, College of Medicine, The Ohio State University, Columbus, OH 43210, USA; Department of Physics, The Ohio State University, Columbus, OH 43210, USA; Steve and Cindy Rasmussen Institute for Genomic Medicine, Nationwide Children's Hospital, Columbus, OH 43205, USA; Pelotonia Institute for Immuno-Oncology, The Ohio State University, Columbus, OH 43210, USA; Department of Pediatrics, College of Medicine, The Ohio State University, Columbus, OH 43210, USA

**Keywords:** melanoma, imaging mass cytometry, mechanistic modeling, stochastic fluctuations

## Abstract

High-dimensional, spatial single-cell technologies, such as CyTOF imaging mass cytometry (IMC), provide detailed information regarding locations of a large variety of cancer and immune cells in microscopic scales in tumor microarray slides obtained from patients prior to immune checkpoint inhibitor (ICI) therapy. An important question is how the initial spatial organization of these cells in the tumor microenvironment (TME) changes with time and regulates tumor growth and eventually outcomes as patients undergo ICI therapy. Utilizing IMC data of melanomas of patients who later underwent ICI therapy, we develop a spatially resolved interacting cell system model that is calibrated against patient response data to address the above question. We find that the tumor fate in these patients is determined by the spatial organization of activated CD8^+^ T cells, macrophages, and melanoma cells and the interplay between these cells that regulate exhaustion of CD8^+^ T cells. We find that fencing of tumor cell boundaries by exhausted CD8^+^ T cells is dynamically generated from the initial conditions that can play a protumor role. Furthermore, we find that specific spatial features such as co-clustering of activated CD8^+^ T cells and macrophages in the pretreatment samples determine the fate of the tumor progression, despite stochastic fluctuations and changes over the treatment course. Our framework enables the determination of mechanisms of interplay between a key subset of tumor and immune cells in the TME that regulate clinical response to ICIs.

**Significance Statement**

Imaging mass cytometry allows for detailed snapshot imaging of microscale organization of tumor and immune cells and provides insights into underlying biology and clinical responsiveness to cancer immunotherapy. Combining published imaging mass-cytometry data and recorded patient responses to immune checkpoint inhibitor (ICI) drugs with analysis rooted in statistical physics and statistical inference theory, we developed and studied the dynamics of mechanistic spatially resolved models. We show that exhaustion of CD8^+^ T cells by melanoma and macrophage cells can lead to protumor fencing of exhausted CD8^+^ T cells around melanoma cells. Specific features of the initial spatial organization can lead to single-variable average behavior despite stochastic spatial dynamics. The insights from our studies can pertain to the response of other solid tumors to ICI therapy.

## Introduction

A tumor is complex and heterogeneous tissue composed of tumor cells, various immune cells, connective tissues, blood and lymphatic vessels, and extracellular materials like collagen (1). The relationship between tumor cells and immune cells in the tumor microenvironment (TME) is shaped by multiple factors, including the recruitment of immune cells (2), activation of T cells by neoantigens in the draining lymph nodes, and exhaustion of activated T cells. Immunotherapeutic strategies that exploit the immune system to eliminate tumors have revolutionized cancer treatment (3). However, despite many striking examples of success, these therapies still cure only a moderate percentage (∼20–40%) of patients (3, 4). System-level understanding of how the immune system induces anti- and protumor responses can considerably aid the development of newer immunotherapies with greater efficacy.

Recent advancements in single-cell spatial technologies, such as CyTOF imaging mass cytometry (IMC) (5–9) and cyclic immunofluorescence (CyCIF) (10, 11), provide extensive information about the composition of the TME where over 30 different proteins can be measured in single cells along with knowledge of their spatial locations in small millimeter scale tissue microarray (TMA) samples extracted from tumors. A wide variety of spatial data analysis tools ranging from calculation of local cell density (12–14), spatial pair correlations (13, 15), clustering (16) to application of probabilistic methods such as latent Dirichlet allocation (15, 17), and machine learning methods such as graph (18) or convolutional (19) neural networks have been employed to identify microscopic spatial patterns of tumor and immune cells in high-dimensional IMC, CyCIF, and multispectral fluorescence imaging datasets that are associated with patient survival and response to anticancer drugs. However, the imaging is usually done at a single time point, mostly before the ICI treatment, and a major challenge is to infer how these microscopic spatial patterns mechanistically determine the tumor growth kinetics when checkpoint drugs are administered.

Spatially resolved mechanistic models involving tumor and immune cells have been developed over the years using agent-based (20–24) and cellular automaton (23) modeling approaches or partial differential equations (25) to determine the roles of interaction between these cell types, vascularization, and mechanical forces in tumor growth and patient responses. These models largely remained uncalibrated against experimental data; however, due to the access of digital hematoxylin and eosin (H&E) slides in recent years, greater computation power, and efficient algorithms, several studies have calibrated spatial models against real (26) and synthetic (27) immunohistochemistry datasets which can identify few types of cells. In contrast, IMC datasets can identify many immune cell types as well as their activation states which offer an attractive scenario to calibrate such spatial models and infer mechanisms involving interplay between the tumor and immune cells.

To this end, we combined IMC data obtained from biopsy samples from melanoma patients precheckpoint therapy, the data regarding the response of these patients to anti-PD1/CTLA4 checkpoint therapy to develop a mechanistic spatially resolved interacting cell system (ICSs) model; we combined statistical interference theory to justify details of the interactions between immune and tumor cells that underlie patient responses to immune checkpoint inhibition (ICI) drugs. The specific major findings from our investigation are as follows: (i) the elucidation of the effects of the interplay between killing of tumor cells by active CD8^+^ T cells, and the exhaustion of the latter by tumor cells and noninflammatory macrophages, (ii) how the interplay in (i) dynamically generates spatial configurations with fencing of tumor cells by exhausted CD8^+^ T cells protecting tumor cells from active CD8^+^ T cells outside the fence; and (iii) how, despite the inherent stochastic fluctuations in the processes, at time scales longer than cell proliferation, maturation and death, specific features in the initial spatial organization of the melanoma and immune cells lead to widely different tumor growth outcomes. Despite the limitations of the dataset due to the small size (1 mm × 1 mm) of the TMA, the small sample size (∼30) of the patient cohort and our model's simplicity, we note that we find and can corroborate the fencing of melanoma cells by exhausted CD8^+^ T cells independently with data from an unrelated experimental study (10). Our modeling approach and methods can be utilized to design new immunotherapeutic strategies for melanoma and other cancers.

### Approach

**Fig. 1.**
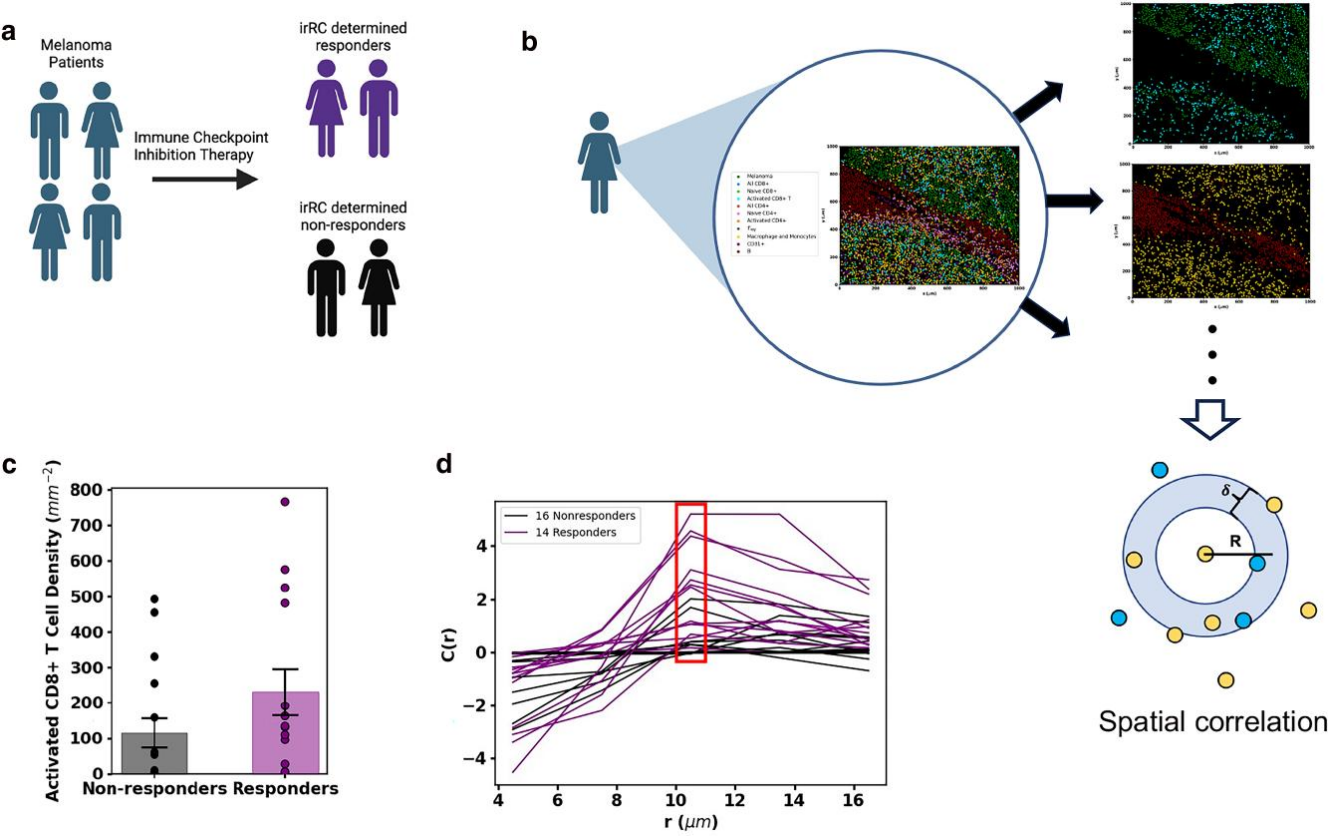
Selection of cell types by analyzing patient slides. a) Patients with melanoma underwent ICI therapy and were characterized as either responders or nonresponders to therapy according to irRC. b) The dataset provides an IMC snapshot of each patient TME. We quantitatively evaluate the relationship between response to therapy and the spatial distributions of each cell type/pairs of cell types. We find the densities of each cell type in every slide as well as the spatial correlation (graphic aid below slides) between every permutation of two cell types. Graphic aid for the spatial correlation calculation: the density of blue cells around each yellow cell at radius *R* in an annulus of thickness *δ* is calculated for each yellow cell and averaged across all yellow cells. The total slide density of blue cells is then subtracted from that average to find if the density of blue cells around the average yellow cell is above or below that expected from a random distribution of blue cells. Finally, this value is divided by the average number of blue cells across all slides. Figures (a-b) were created with BioRender.com. c) The activated CD8^+^ T-cell density in slides corresponding to patients who did or did not respond to ICI therapy. The average activated CD8^+^ T-cell density (represented with box) among responders is higher than that of the nonresponders. The averages are different with *P*-value 0.1504. Because activated CD8^+^ T densities differ between responders and nonresponders, we consider activated CD8^+^ T cells a relevant cell population. d) The plot of spatial correlation between macrophages/monocytes and activated CD8^+^ T cells shows that at a distance of 10.5 μm (about the distance to a nearest neighbor), macrophages/monocytes find on average more activated CD8^+^ T-cell neighbors in responders than in nonresponders. The average spatial correlation values at 10.5 μm are different with a *P*-value of 0.0005. This difference between responders and nonresponders to ICI in the spatial distribution shows the relevance of the cell types, macrophages/monocytes and activated CD8^+^ T cells, and their spatial distribution.

We obtained publicly available CyTOF IMC data of 1 × 1 mm^2^ TMAs to quantify proteins in single cells that yield a spatial profile of different cell types in the TME. This information for samples from a cohort of 30 melanoma patients is paired with the response (responder or nonresponder, determined by immune-related response criteria or irRC (28)) to immune checkpoint inhibitors (ICIs) involving anti-PD1, anti-CTLA4, or the combination of the two (Fig. 1a) (5, 28).

We followed three main steps. First, we used the IMC and the patient response datasets to identify the immune cell types and their microscale spatial organization that are associated with the response of patients to ICI drugs. Second, we developed a spatially resolved ICS model involving mechanistic interactions between tumor and the relevant immune cell types identified in the previous step. Lastly, we set up a training and testing framework for the spatially resolved mechanistic model using the IMC and the patient response datasets; this allows us to evaluate different hypotheses regarding the interactions underlying the interplay between tumor and immune cells. We describe these steps briefly below and provide further details in the Materials and methods section and in the Supplementary material.

#### Identification of relevant spatial microscale cellular patterns

We used the spatial data regarding the locations of 10 different cell types such as melanoma cells, activated CD8^+^ T cells, and macrophage/monocytes in the TMA slides that were determined by Moldoveanu et al. (5) for our analysis (Fig. 1). First, we computed the densities of these different cell types in each TMA slide, which displayed large patient–patient variations. To determine cell types whose densities differ substantially between responders (14 patients) and nonresponders (16 patients) of the ICI therapy, we compared the mean densities of the cell types averaged over TMA slides obtained from responder or nonresponder patients (Fig. S1a). We found that responders have on average larger densities of activated CD8^+^ T cells (*P* = 0.16; Fig. 1c); however, the densities of all the cell types did not display substantial difference (*P* ≤ 0.05) between the responder and nonresponders (Fig. S1a).

Next, we evaluated whether specific microscale organization of certain cell types in the TMA slides significantly separates responders from nonresponders. We used the pair correlation function (*C*(*r*)), a widely used approach in statistical physics and material science (29), to evaluate whether cells of the same type or different types cluster or avoid each other within a length scale *r* relative to homogeneous and random spatial distribution of the cells in the slide (Fig. 1b and d). We computed *C*(*r*) for all the cell types and all possible pairs of the cell types, which showed large patient–patient variations (Figs. S1b and 1b). In order to determine spatial patterns involving specific cell types that might distinguish responders to nonresponders, we compared values of *C*(*r*) at *r* = 10.5 μm for all possible pairs of cell types and found that *C*(*r*) for macrophages/monocytes with activated CD8^+^ T cells differ substantially (*P* = 0.002) between responders and nonresponders, whereas the *C*(*r*) for most of the other pairs of cell types cannot be well separated (*P* > 0.05, Fig. S1b). The only cells other than CD8^+^ T cells and macrophage/monocytes that showed *C*(*r*) values substantially (*P* = 0.049) different between responders and nonresponders are CD4^+^ T cells and stromal endothelial CD31^+^ cells. Stromal and immune cell interactions can influence tumor growth and metastasis via cytokines and tumor cell differentiation (30). In this study, we focused on the growth and lysis of tumor cells by CD8^+^ T cells, leaving the influence of stromal and immune cells in tumor growth as a future direction. Based on the above analyses, we reasoned that melanoma cells, activated CD8^+^ T cells and macrophage/monocytes, and mechanisms involving interactions between these cell types give rise to the differences in tumor growth in the patients who went through the ICI therapy. In order to evaluate the roles of different mechanisms with which these cells can interact to regulate tumor growth, we developed a spatially resolved mechanistic ICS model as we describe below. More information on the spatial analysis can be found in the Analysis of IMC datasets section in Supplementary material.

#### Development of a spatially resolved ICS model

**Fig. 2.**
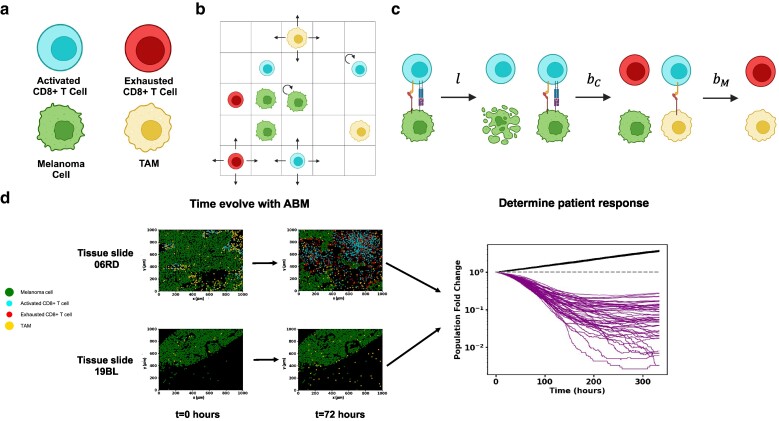
Schematic of model and approach to parameter estimation and hypothesis testing. a) The four cell types in the model: including melanoma cells, activated CD8^+^ T cells, exhausted CD8^+^ T cells and TAMs. b) Schematic depicting melanoma cells, macrophages, activated CD8^+^ T cells, and exhausted CD8^+^ T cells on the ICS lattice. CD8^+^ T cells and macrophages are free to diffuse around the slide. c) Cell–cell interactions in the model include lysis of melanoma cells by activated CD8^+^ T cells at rate *l*, exhaustion of activated CD8^+^ T cells by melanoma cells at rate $b_{C}$ and exhaustion of activated CD8^+^ T cells by TAMs at rate $b_{M}$. Rates and rules are given in Table S1. Figures (a), (b) and (c) were created with BioRender.com. d) Shows spatial locations of melanoma cells, macrophages, cytotoxic CD8^+^ T cells, and exhausted CD8^+^ T cells from IMC data of human melanoma biopsies taken before ICI treatment and corresponding computationally time-evolved samples after 2 days. Top: IMC slide 06RD corresponding to a responder. Bottom: IMC 19BL corresponding to a nonresponder. Right: Melanoma cell population trajectories in time for 50 samples for each of the 2 slides. The 19BL samples display cancer growth showing nonresponse, and the 06RD samples display cancer regression, which is an example of response.

We developed a spatially resolved model to describe the time evolution of melanoma cells and specific immune cells identified through our data analysis. The model is set up on a 1 × 1 mm^2^ 2D simulation box divided into smaller *l*_0_ × *l*_0_ (=10 × 10 μm^2^) chambers. We considered activated CD8^+^ T cells, exhausted CD8^+^ T cells, tumor-associated macrophages (TAMs), and melanoma cells (Fig. 2a), where these cells interact and move spatially with specific rules and proliferate, die, or differentiate. The rules are based on experimental observations reported in the literature or on previous computational modeling efforts. We briefly describe the rules used in the model below. We also provide biological justifications for the rules and parameter values associated with the rules in Table S1 as well as in the Model simulation section in Supplementary material.

In the model, all four cell types can occupy chambers in the simulation box, obeying local occupation limits due to the cells' physical sizes. Melanoma cells proliferate (Fig. 2b) and are lysed by activated CD8^+^ T cells when the melanoma cells are in contact with the CD8^+^ T cells in the same or neighboring chambers (Fig. 2c). When in contact with melanoma cells, activated CD8^+^ T cells can become exhausted. Activated CD8^+^ T cells proliferate, creating new activated CD8^+^ T cells in the same chamber. Activated CD8^+^ T cells are also recruited into a randomly chosen chamber. The rates of proliferation and recruitment of activated CD8^+^ T cells depend on the number of melanoma cells lysed within a time interval. The exhausted CD8^+^ T cells do not lyse melanoma cells or proliferate, and die. TAMs, similar to melanoma cells, induce CD8^+^ T-cell exhaustion when those are in contact with activated CD8^+^ T cells in the same chamber. Spatial motility of CD8^+^ T cell and TAM is modeled as diffusive hops to the nearest neighboring chambers. We do not explicitly model subcellular kinetics such as the pharmacodynamics of anti-PD1 and anti-CTLA4 for simplicity but the effects of ICI drugs are implicitly captured in model parameters. More details are given in the Model simulation section in Supplementary material and Table S1.

#### Modeling the time evolution

We approximate the randomness in cell movements, cell proliferation, cell death, and cell differentiation events as Markov processes, where the current state only depends on the previous state of the system, for simplicity. The time evolution is performed by a kinetic Monte Carlo simulation approach (for details, see Model simulation section of Supplementary material).

#### Model training

The time evolution of our model depends on the values of the model parameters. The order of magnitudes of many of the parameters are known from previous experiments and modeling efforts; however, some of the parameters that substantially affect the tumor growth, such as the rate of exhaustion of activated CD8^+^ T cells by melanoma cells or macrophages, are not known. We estimated these parameters using the patient response outcomes in the following way: The initial locations and numbers of the melanoma and immune cells in our model are obtained from the TMA slides, and the locations and numbers of these cells change as the model is evolved over time. We computed the fold change of the total number of melanoma cells from the initial configuration at *t* = 0 with that of at an end time *T*, where a fold change of <1 (or >1) designates the patient associated with the TMA slide as a responder (or nonresponder); Fig. 2d. We choose *T* = 333 h or about 2 weeks which spans a common duration of a single cycle of ICI therapy through the course of a full ICI therapy regimen (7, 31, 32). We then assume that the effects of ICI remain constant throughout the cycle and that outcome for a single cycle of treatment correlates with patient treatment outcome determined by Moldoveanu et al. using irRC. Since the time evolution is stochastic, the above fold change can vary across multiple simulations of the same initial condition with the same model parameters. We therefore define a *prediction success frequency f_i_*(θ) for each slide *i* to be the fraction of the simulations of our ICS model with parameter set ***θ***, given the initial condition fixed by slide *i*, which agrees with the reported binary clinical outcome (e.g. responder or nonresponder) for the corresponding patient. We then define an appropriate response prediction success score function score(***θ***) (Eq. 2) for the model to quantify the model's success across all slides. The score function given the clinical outcome data is maximized to estimate the model parameter set ***θ***. We may then simulate the cancer cell population progression for each patient slide at optimal model parameters (Fig. S6). Additional details are provided in the Materials and methods section.

#### Hypothesis testing

**Fig. 3.**
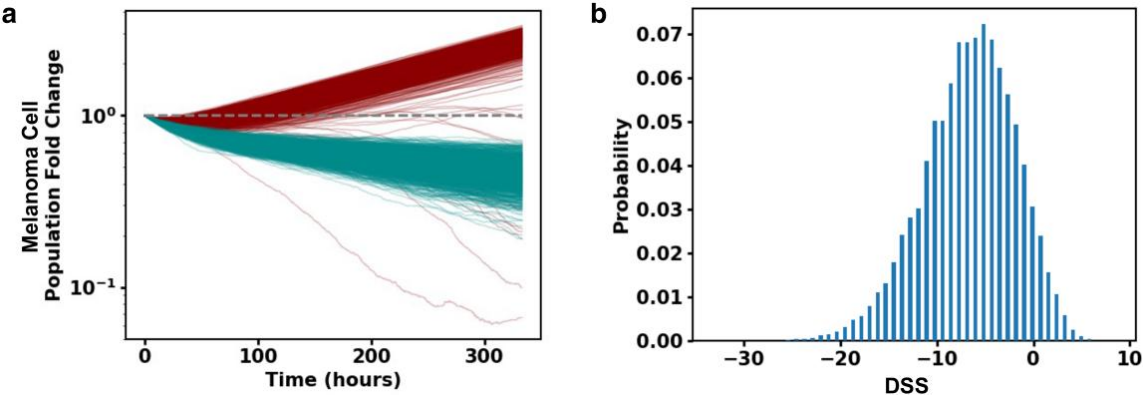
Hypothesis testing with ICS model and patient response data suggests TAM and melanoma cell–induced exhaustion of CD8^+^ T cells regulate response to ICI drugs. a) Melanoma cell fold change (log-linear plot) trajectories plotted as a function of time for 1,000 simulations of slide 33RD performed with the trained full model (dark red) and with the trained model with TAM exhaustion of activated CD8^+^ T cells turned off (dark cyan). b) Shows the distribution of DSS (defined in the Materials and Methods section) over 100,000 bootstrap samples to test the hypothesis that the prediction power of the full model is the same as in the model without TAM exhaustion of activated CD8^+^ T cells. The hypothesis is rejected with *P*-value 0.070. DSS distribution for the model without exhaustion of CD8^+^ T cells by melanoma cells is shown in Fig. S2.

We set up a hypothesis testing framework for evaluating different mechanisms that can potentially underlie the regulation of tumor growth by the immune cells considered in our ICS model using model simulations, IMC datasets, and the clinical outcome data. Alternate hypotheses (e.g. TAMs do not exhaust activated CD8^+^ T cells) can be tested by setting model parameters in the base model (the model described up to this point) to zero to create alternate models. To compare these alternate models to the base model, we first train the alternate models with the clinical outcome data as we trained the base model (Fig. 3a). Once these alternate models are trained, we then utilize bootstrapping to generate new datasets. Finally, comparing the prediction success scores (or *score*(***θ***)) for each dataset between the base model and a given alternate model, we evaluate whether the alternate model is more successful at predicting response (Figs. 3b and S2). Further details are provided in the Materials and methods section.

## Results

### Exhaustion of CD8^+^ T cells induced by TAMs and melanoma cells regulate response to ICIs

We tested several hypotheses pertaining to the mechanistic interplays between CD8^+^ T cells, TAMs, and melanoma cells that have been previously explored in experimental investigations with animal models, and in vitro and ex vivo studies by applying our approach to the IMC and the patient response data in Moldoveanu et al. (5). To test whether macrophages play a protumor role in the TME, we evaluated the hypothesis that exhaustion of CD8^+^ T cells by TAMs and melanoma cells plays a protumor role and influences the response to ICI drugs in patients. We compared the base model with alternate models (representing hypotheses) where either the rates with which TAMs or melanoma cells exhaust CD8^+^ T cells when these cells interact is set to zero. The absence of CD8^+^ T-cell exhaustion by either melanoma cells or TAMs in the alternate models led to larger decreases in the fold change of the melanoma cells compared with that in the base model (Fig. 3a). This is due to the presence of more activated CD8^+^ T cells in the TME which sustained cytotoxicity toward the melanoma cells for the duration of the simulation. We evaluated the consequence of removing exhaustion of CD8^+^ T cells by melanoma cells and TAMs as in the alternate models on their ability to predict response to ICI drugs within our hypothesis testing framework. The *P*-values obtained were slightly higher (>5 and <10%), usually denoted as suggestive (33), than the community accepted value (≤5%). We found suggestive *P*-values of 7% (TAM exhaustion of CD8^+^ T cells to zero) and 9% (melanoma cell exhaustion of CD8^+^ T cells to zero), respectively, rejecting the alternate hypotheses; therefore, the alternate models are less successful in predicting the patient outcome compared with the base model (Figs. 3b and S2). Thus, this result points to the importance of the exhaustion of CD8^+^ T cells by TAMs and melanoma cells in regulating tumor growth and response to ICI drugs in melanoma.

### The initial (pretreatment) spatial organization of immune cells determines melanoma cell growth in the TME

Characterization of the trained ICS dynamics yields insight into how the TME might evolve in vivo. In particular, we find that the initial spatial distribution of activated CD8^+^ T cells and TAMs in the ICS model impacts the average dynamics over all simulations (trajectory average) of melanoma and activated CD8^+^ T cells for several patient slides. To evaluate the role of the initial spatial distribution of the immune cells in the TME, we first uniformly and randomly distributed activated CD8^+^ T cells or TAMs throughout the area of each slide to generate altered initial distributions of the immune and melanoma cells. We then investigated the ICS time evolutions of the altered initial distributions and their ability to predict the patient responses associated with those slides. We describe two such cases below.

**Fig. 4.**
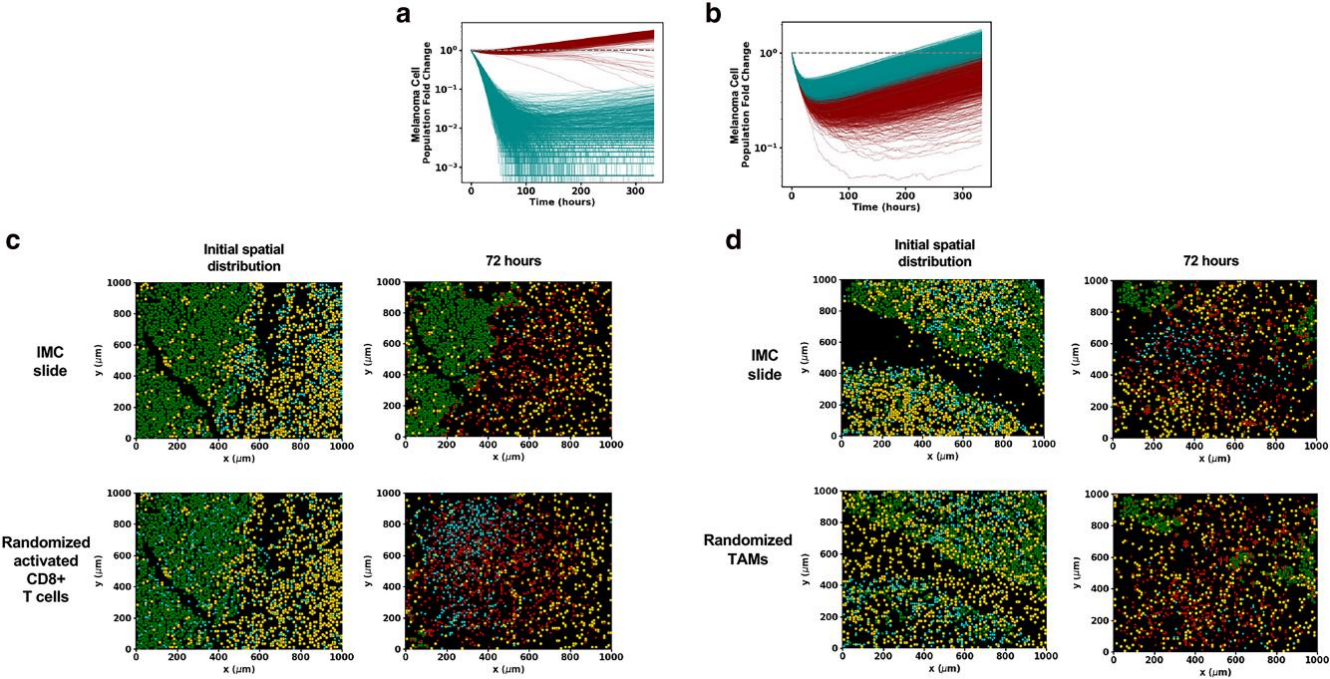
The pretreatment spatial organization of activated CD8^+^ T cells, TAMs, and melanoma cells determines tumor cell dynamics in the ICS model. a) Melanoma cell fold change as a function of time on a log-linear plot for 1,000 samples of slide 33RD. Contrast the curves for the observed ICI initial condition shown in dark cyan with those for the same initial condition but with the initial activated CD8^+^ T-cell positions randomly distributed (shown in dark red). With the rearrangement of the activated CD8^+^ T cells, the model prediction flips from nonresponse to response in almost all runs of the stochastic simulations. b) Similar comparison of melanoma cell fold change for 1,000 samples of slide 21RD as a function of time; the results for the observed ICI initial condition are shown in dark red, and the results with only the initial TAM positions being randomly distributed are shown in dark cyan. The randomization of the initial TAM positions changes the behavior from responder to nonresponder for almost all samples. c) Snapshots of a time evolution of slide 33RD, considered in (a), for one sample using the ICS model (top) and for the same initial condition but with the activated CD8^+^ T-cell positions randomly distributed (bottom). When the activated CD8^+^ T-cell population is randomized, it is distributed throughout the melanoma bulk and is thus able to rapidly expand due to its early lysing of melanoma cells. d) Snapshots from a single-time evolution of slide 21RD considered in (b) using the ICI initial condition (top) and of the same initial condition but with the TAM spatial distribution randomly distributed (bottom). The positions of the randomized TAM cells can more effectively inhibit the expansion of the activated CD8^+^ T-cell population leading to a different outcome. Results for figures (a)–(d) were obtained with the trained base ICS model. The color scheme separating cell types in the tumor slides (c) and (d) are the same as those used in Figure 2a.

Consider slide 33RD corresponding to a patient who did not respond to ICI therapy. The base model showed a net increase in the average number of the melanoma cells at the final time (about 14 days) and correctly predicted the patient response in 98.6% of 1,000 simulations performed of the slide TME (Fig. 4a). However, when the initial spatial distribution of the CD8^+^ T cells was altered by seeding the cells uniformly and randomly throughout the slide, there was a net decrease in the total number of melanoma cells over the duration of the simulation, and 100% of the 1,000 simulations of the altered initial condition incorrectly predicted the slide to be a responder (Fig. 4a). Inspection of the spatial organization of the melanoma, TAMs, and activated CD8^+^ T cells in slide 33RD (Fig. 4c), shows that a large portion of the melanoma and the activated CD8^+^ T cells are spatially segregated, which could limit the access of the CD8^+^ T-cell population to the majority of the melanoma cells, whereas when the activated CD8^+^ T cells are distributed homogeneously in the altered initial distribution, most of the melanoma cells can be accessed and eliminated by the CD8^+^ T cells. This leads to a net decay of the melanoma cell population and causes the model to predict the slide to be a responder. Similarly, randomizing the initial distribution of activated CD8^+^ T cells in the rest of the patient cohort yields largely different model predictions (a shift in the percentage accuracy of trajectory prediction of patient response >60%) for two other patient slides (10% of patient slides) as well (Table S3).

In another slide, 21RD, associated with a responder, the time evolution of the initial distribution of the melanoma and immune cells using our ICS model correctly predicts the outcome 98% of the 1,000 simulations (Fig. 4b). However, when the spatial distribution of the TAMs in slide 21RD is altered by seeding the TAMs randomly and uniformly throughout the slides, only 65 of 1,000 simulations show a decrease in the melanoma cell population by final time thus predicting the correct outcome 0.065% times (Fig. 4b). For the slide 21RD, distributing the TAMs homogeneously throughout the slide (Fig. 4d) increases mixing between the TAMs and activated CD8^+^ T cells which increases conversion of exhausted CD8^+^ T cells and, ultimately, leads to increased growth of the melanoma cell populations and incorrect response prediction for the patient. Clearly, ICI response associated with a slide is dependent on specific features of the initial spatial distribution of cells in the TME. Randomizing the initial distribution of TAMs in the rest of the cohort yields largely different model predictions for one other patient slide (∼7% of patient slides; Table S3). These results point to potential mechanisms with which the initial spatial distributions of the melanoma and immune cells can regulate the tumor growth in the presence of ICI drugs. We further characterize and quantify such spatial mechanisms in the next two sections.

### Intratumoral CD8^+^ T cells produce fencing of tumor boundaries with exhausted CD8^+^ T cells

**Fig. 5.**
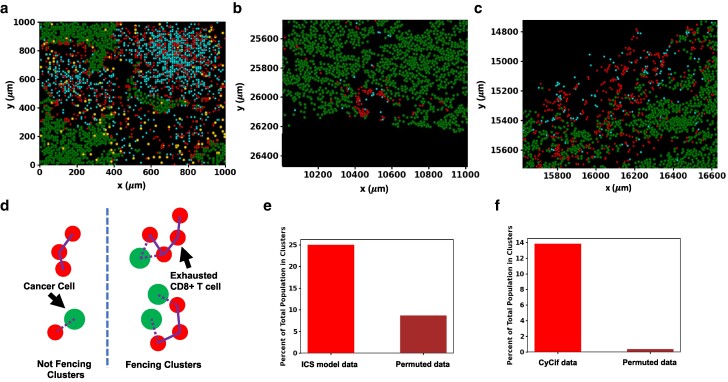
Fencing of tumor boundaries with exhausted CD8^+^ T cells in the ICS model and CyCIF imaging data of TMA slides from melanoma patients. a) A sample of slide 06RD at 72 h of simulation shows exhausted CD8^+^ T-cell fencing. b, c) All melanoma, cytotoxic CD8^+^ T cells and exhausted CD8^+^ T cells are plotted for a subsection of slide MEL06 (10). Exhausted CD8^+^ T-cell fencing in the model is supported by the presence of linings of exhausted CD8^+^ T cells along the boundary of the tumor in MEL6 blocking access to their cytotoxic counterparts. In general, exhausted CD8^+^ T cells border the melanoma cells more often than cytotoxic CD8^+^ T cells. This may lead to a reduction of cytotoxic function in vivo. d) Illustration of fencing and nonfencing clusters with exhausted CD8^+^ T cells and melanoma cells. The two clusters of exhausted CD8^+^ T cells on the left, considered to be nonfencing, are either not in contact with melanoma cells or do not have enough cells in the cluster. The two clusters on the right meet the criteria for being fencing clusters. e) Shows the fencing metric for ICS model data alongside that for randomly a permuted distribution of exhausted CD8^+^ T cells. When the spatial distribution of exhausted CD8^+^ T cells is randomly permuted with all CD8^+^ T cells and TAMs in the simulation the percentage of exhausted CD8^+^ T cells contributing to fencing in slide 06RD at 72 h is considerably reduced when compared with the actual distribution. f) Shows the fencing metric for the CyCif data alongside that for randomly a permuted distribution of exhausted CD8^+^ T cells. The percentage of all exhausted CD8^+^ T cells contributing to fencing in slide MEL06 is similarly greatly reduced when the distribution of exhausted CD8^+^ T cells is randomly permuted with all identified cells except melanoma cells. The color scheme separating cell types in the tumor slides (a), (b) and (c) are the same as those used in Figure 2a.

We studied the spatial configurations of the melanoma and the immune cells in the ICS model starting from each of the slides to identify features that may potentially affect tumor dynamics. We found that the system displays an increased accumulation of exhausted CD8^+^ T cells in contact with melanoma cells, which we term “fencing.” This aggregation of exhausted CD8^+^ T cells in contact with melanoma cells occurred for simulations initialized with several IMC slides (Fig. 5a). The phenomenon of “fencing” occurred not only in the snapshot configurations shown but persisted on a time scale comparable to (or larger than) that is set by the melanoma cell proliferation rate. We quantified the aggregation of the exhausted CD8^+^ T cells in contact with melanoma cells in our simulations. We present the analysis in the context of slide 06RD and show results for additional slides in the Supplementary material (Fig. S3a and b). We defined a collection of exhausted CD8^+^ T cells in contact with melanoma cells as a fencing cluster of exhausted CD8^+^ T cells where at least one exhausted CD8^+^ T cell in the cluster shares the same chamber or a nearest neighboring chamber with a melanoma cell and all the others are in contact with fellow exhausted CD8^+^ T cells in the same chamber or the nearest neighboring chambers (Fig. 5d). The shape of the clusters of exhausted CD8^+^ T cells may not always resemble the canonical shape of a fence. We found that for the simulations initiated with the slide 06RD, for configurations at 72 h, 25% of the exhausted CD8^+^ T cells reside in such clusters containing at least three exhausted CD8^+^ T cells (Fig. 5e). In contrast, when the CD8^+^ T cells in these configurations were permuted randomly with other immune cells (activated CD8^+^ T cells and TAMs), only 9% of the randomized exhausted CD8^+^ T cells contribute to the clusters with three or more cells, implicating roles of the spatial distributions of the melanoma and the immune cells in the IMC slides and their kinetics in giving rise to these fencing structures. In our simulations, the fencing structures arise because of the exhaustion of active CD8^+^ T cells in contact with melanoma cells. The exhausted CD8^+^ T cells that can no longer lyse the melanoma cells in the fence hinder access of other activated CD8^+^ T cells to the tumor, thus potentially reducing lysis. We find evidence of fencing cluster formation by exhausted CD8^+^ T cells in the simulation of 20 of the 30 patient slides (Table S4). Given the generality of such a mechanism underlying the occurrence of fences of exhausted CD8^+^ T cells, we reasoned these structures should be seen in images of tumor samples of melanoma.

We investigated CyCIF imaging data of melanoma tissue slides obtained from patients at different stages of melanoma progression technique published in a different study by Nirmal et al. (10). The CyCIF imaging data contained over 50 protein markers and identified over 10 different types of immune, stromal, and melanoma cells, including melanoma and exhausted CD8^+^ T cells. We found fencing structures formed by exhausted CD8^+^ T cells in several regions (two are displayed in Fig. 5b and c). In analyzing the CyCIF data, we considered the typical physical sizes of the cells in the system, by modifying our definition of nearest neighbors to compute the clusters of exhausted CD8^+^ T cells in contact with melanoma cells (Fig. 5d). Here, two cells are considered nearest neighbors if their nuclei are within 15 μm of each other. The results of our analysis of slide MEL-6 from Ref. (10) are as follows: 14% of exhausted CD8^+^ T cells contribute to fencing clusters with three or greater number of member cells; randomly permuting the spatial distribution of the same exhausted CD8^+^ T cells, we found that 0.05% are in the fencing clusters, eliminating the possibility that the fencing occurs in random configurations (Fig. 5f). This calculation was also performed with neighbors being defined at other radii, and the same qualitative results were found. These results show that exhausted CD8^+^ T cells preferentially cluster near melanoma cells in vivo, similar to that observed during the time evolution of the ICS model.

### Growth of melanoma cells in the TME is regulated by stochastic fluctuations

In this section, we present results to show how the interplay of the initial spatial distribution of cell types from patient data with stochastic dynamics of the ICS model determines the kinetics of the number of melanoma cells (Fig. S4a). We will illustrate results from our investigations using two slides as examples, examine their implications, and make several general conclusions.

**Fig. 6.**
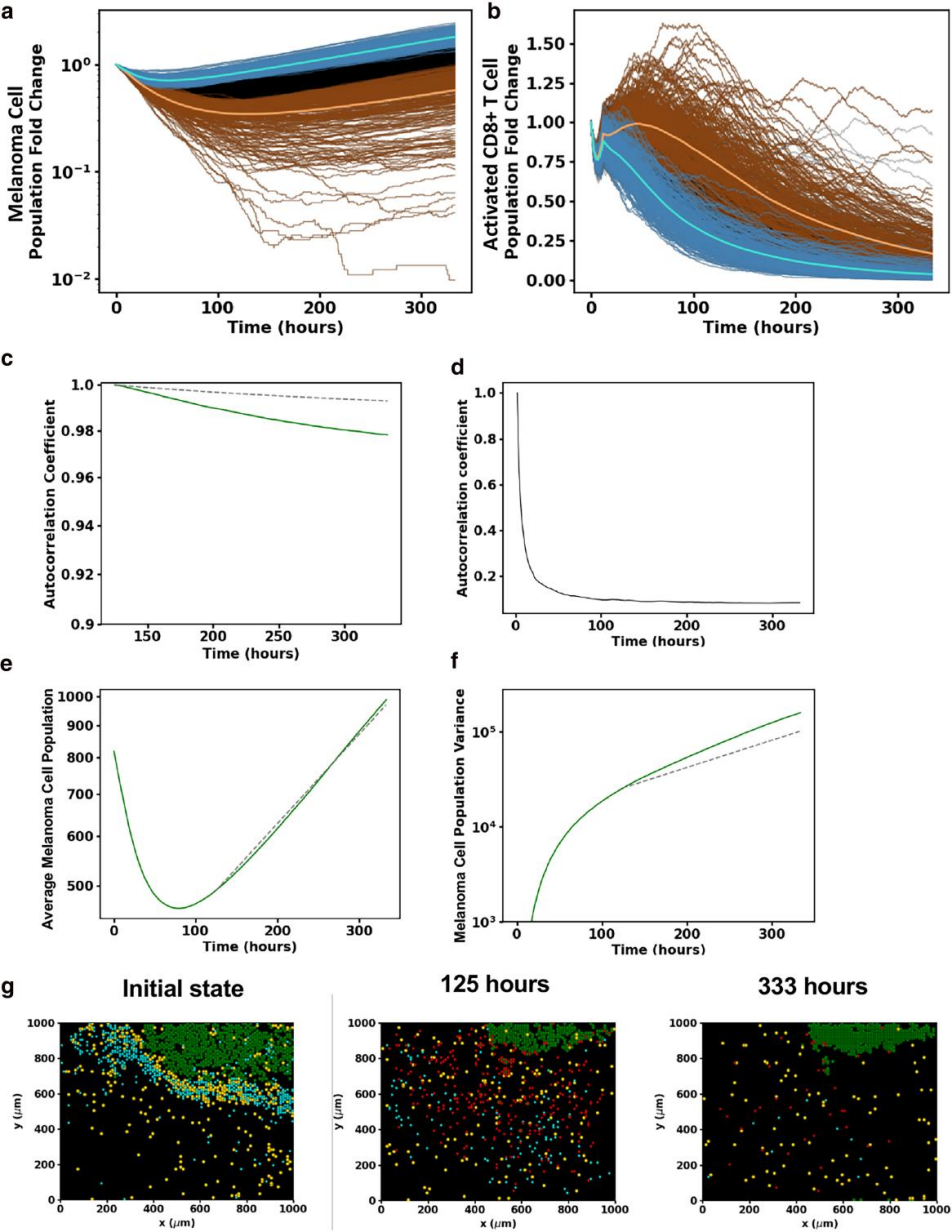
Characterization of the interplay between stochastic fluctuations and initial spatial organization of CD8^+^ T cells, TAMs, and melanoma cells in regulating tumor growth in the ICS model. a) Shows temporal stochastic trajectories of the populations of melanoma cell (log-linear plot) and b) activated CD8^+^ T-cell population (linear–linear plot) for 1,000 different runs of the same initial distribution of the cells in slide 16BL. Ordering the trajectories by their melanoma cell populations at 125 hours, the trajectories corresponding to the top 25% highest (lowest) melanoma cell populations are blue (brown). The lighter blue (brown) trajectory corresponds to the average trajectory of the blue (brown) trajectories. The black trajectories represent the remaining 50% of trajectories which are neither in the blue nor brown subgroups. All samples begin with identical initial conditions set by the patient slide data 16BL. Observe that the samples in the top 25% and bottom 25% of the melanoma cell population identified at 125 h remain separated up to the final time. c) The change in the autocorrelation coefficient (Eq. 1) with time for the melanoma cells from 125 h of the samples shown (solid line) along with the analytically computed autocorrelation of a single-variable stochastic birth process (dashed line). Both these autocorrelation coefficients decrease with time very little in the final 175 h of the simulation. d) Shows the change in the autocorrelation coefficient of the melanoma cells in the ICS model with time from 2 h to final time. This shows how predictive ability of the future state starting from 2 h drops off very fast in the early stage. After 125 h, the autocorrelation coefficient drops off very little as in (a). e) A log-linear plot of the mean melanoma cell population as a function of time from the simulations compared with the fitted single-variable growth process from 125 h. f) A log-linear plot showing the change of the melanoma cell population variance from the simulations (solid line) compared to that from the single-variable growth process (dashed line) from 125 h. The ICS model shows good qualitative agreement with the single-variable growth model. The variance is larger for the ICS model, as there are still some spatial dependences that contribute to variations in population. g) Snapshots of the spatial distribution of the activated and exhausted CD8^+^ T cells, TAMs, and melanoma cells from a single-time evolution of slide 16BL using the ICS model. The melanoma and activated CD8^+^ T cells become spatially isolated as the melanoma cell population dynamics transition to the later growth stage. This isolation persists up to the final time as the activated CD8^+^ T cells are exhausted, and the melanoma cell population proliferates mimicking a stochastic birth process. The color scheme separating cell types in the tumor slides (g) are the same as those used in Figure 2a.

We first examined the kinetics exhibited when the initial condition corresponds to slide 16BL (Fig. 6a and b). The stochastic trajectories describing the fold change of the melanoma cell number over time fluctuate and can be seen to cross at early times (*t* ≤ τ* ∼125 h for 16BL), whereas, as time progresses (e.g. >125 h), the overwhelming majority of the 1,000 trajectories (except for a few dozen outliers) remain separated (Fig. S4c).

We further characterized the crossing and noncrossing of most of the stochastic trajectories at early and late times (roughly before and after 125 h), respectively, using an autocorrelation coefficient function $A(t,t_{i})$,


(1) $$\;A(t,t_{i})=\frac{1}{N}\underset{\alpha \varepsilon \;\mathrm{all}\;\mathrm{samples}}{\sum }\frac{\left(C_{\alpha }(t_{i})-\mu (t_{i}))(C_{\alpha }(t)-\mu (t)\right)}{\sigma (t_{i})\sigma (t)}.$$

where *N* is the number of samples (stochastic trajectories), $\mu (t)=\frac{1}{N}\sum_{\alpha \varepsilon \;\mathrm{all}\;\mathrm{samples}}C_{\alpha }(t)$, and $\sigma^{2}(t)=\frac{1}{N}\sum_{\alpha \varepsilon \;\mathrm{all}\;\mathrm{samples}}{\left(C_{\alpha }(t)-\mu (t)\right)}^{2}\;$. $A(t,t_{i})$ quantifies the correlation between the populations at the current time *t* and an earlier time $t_{i}$. The results of the simulations for the slide 16BL are shown in Fig. 6c and d; they show a sharp decay of $A(t,t_{i}=2\;h)$ with *t* for *t* > 2 h capturing the lack of correlation with the initial state or mixing of the stochastic trajectories due to stochastic dynamics, whereas $A(t,t_{i}=125\;h)$ decays slowly showing the decreased mixing of the stochastic trajectories.

The late-time kinetics of the mean fold change for the trajectories beyond τ* can be well approximated by an exponential growth with a proliferation rate roughly 25% lower than that of the melanoma cell replication rate. The decrease can be attributed to the elimination of the melanoma cells by the remaining small population of activated CD8^+^ T cells that is still decreasing due primarily to TAM exhaustion. The decreased mixing of the trajectories for *t* ≥ τ* is reflected in the coefficient of variation (*σ*/*μ*, Fig. S4b); it plateaus around a value of 0.4 at late times while the mean melanoma population *μ* increases exponentially.

The above two observations suggest that the dynamics of the melanoma cell number can be captured by a single-variable random birth process, known as the Yule process (34). Fitting the birth rate, the only parameter in the Yule model, to the observed growth rate in the simulations, we compared the analytical expression of the variance of the Yule process with that in the simulations (Fig. 6e). The one-variable model variance is somewhat lower than that in the simulations (Fig. 6f). This difference between the ICS model variance and the Yule process can be understood by noting the spatial fluctuations in the number of activated CD8^+^ T-cell population that reduce the effective growth rate. The autocorrelation coefficient function $A(t,t_{i}=125\;h)$ for the Yule process agrees with that of the ICS model. The fact that we can describe the late stage of the ICS model evolution by a spatially independent model is a striking feature of the model. Next, we examined the qualitative mechanism that leads to the change in the behavior of melanoma cell population, around 125 h, that is characterized by a rapidly decaying autocorrelation coefficient function at early times (Fig. 6d) and a slowly decaying autocorrelation coefficient function at late times (Fig. 6c). We reasoned this is due to a spatial separation between the melanoma and the CD8^+^ T-cell populations at late times (≥125 h). Inspection of the spatial configurations at intermediate times (Fig. 6g), revealed that around 125 h when the average melanoma cell population is near its minimum, the melanoma cells are spatially separated from the activated CD8^+^ T-cell population. The activated CD8^+^ T cells diffuse through the region with a small probability for encountering and eliminating melanoma cells and only rarely contact the isolated cancer masses. This spatial separation underlies the success of the single-variable model at late times.

At early times when the CD8^+^ T cells in closer contact with melanoma cells the lysing of the melanoma cells leads to greater proliferation and recruitment of CD8^+^ T cells that compensates the exhaustion by TAM and melanoma cells leading to continued decrease of the melanoma cell population. The effectiveness of the feedback mechanism depends on the spatial disposition of the cells in the initial slide and at early times and determines whether the spatial separation occurs and if so when. For some of the slides, this spatial segregation occurs at early times as in 16BL and similar descriptions are valid (Fig. S6). On the other hand, if the diverse types of cells remain heterogeneously distributed as time progresses, the feedback persists and there is a decrease in the average number of melanoma cells up to late times and even up to the final time as in slide 06RD (Fig. S5a–c) which exhibits mixing as shown by its autocorrelation (Fig. S5d). We analyzed stochastic trajectories in other slides which help us make a few general observations described below.

We may break the stochastic dynamics of the other slides into mixing and lower mixing or dispersed trajectory stages similarly to 16BL. The dynamics in all slides except for those with small initial melanoma cell populations may be described by either mixing stages, dispersed stages, or with a transition from the mixing trajectory stage to the dispersed trajectory stage before final time (Fig. S5). For the slides giving rise to stochastic kinetics with the dispersed trajectory stage, the initial activated CD8^+^ T-cell population count and the initial spatial distribution of activated CD8^+^ T cells determine the time and melanoma cell population count when the slide dynamics transition to the dispersed state. Slides that maintain the mixing stage to final time are characterized by larger activated CD8^+^ T-cell populations at late times.

## Discussion

We integrated cell-level, CyTOF IMC dataset and patient-level response data to ICI treatment in melanoma to develop a mechanistic, spatially resolved ICS model; we developed a statistical framework to justify and calibrate interactions between melanoma, CD8^+^ T cells and TAMs in the microscale in the TME. Our simulations allow us to predict responses to ICI treatment and the spatiotemporal development of the TME starting from the snapshot IMC datasets. We used the ICS model to elucidate how specific changes in the spatial co-existence of melanoma and immune cells in the initial state lead to dynamic changes in the spatial organization of the melanoma and the immune cells on microscopic length scales and thus, dramatically affect the growth of the melanoma cell populations. A unique aspect of our model development is the way in which we determined the relevant cell types in our mechanistic ICS model using the IMC and the patient response data, and further estimated model parameters in our model. Furthermore, we studied the time evolution starting from the initial condition provided by the imaging data. This enabled us to identify the important spatial features in much lower dimensions (e.g. three cell types) within the large number of cell types, cytokines, and chemokines that compose the TME that are crucial in determining the tumor fate.

Inspection of the spatial patterns generated during our ICS simulations initiated from several TMA slides showed the emergence of a fencing pattern of exhausted CD8^+^ T cells around tumor boundaries. The fencing patterns arise as activated CD8^+^ T cells encounter melanoma cells and become exhausted. The exhausted CD8^+^ T cells in the fencing patterns prevent activated CD8^+^ T cells from invading the interior of the tumor and can potentially protect the tumor cells from CD8^+^ T-cell cytotoxicity. We found confirmatory evidence for such fencing structures formed by exhausted CD8^+^ T cells (show the biomarkers, e.g. TIM3^+^ and Lag3^+^) in CyCIF imaging data of TMAs obtained from melanoma patients by Nirmal et al. (10). This also suggests the underlying mechanism leading to these patterns in our simulations may be operative in melanoma patients. The exhausted CD8^+^ T cells in the data by Nirmal et al. are likely to arise due to PDL1-PD1 axis–induced exhaustion by TAMs and melanoma cells. It will be important to determine whether the similar mechanism underlying the fencing pattern of exhausted CD8^+^ T cells is present in solid tumors other than melanoma, and the differences in the subtypes of CD8^+^ T cells residing within and outside the fencing structures.

Our investigation found that the spatial segregation of melanoma cells and activated CD8^+^ T cells plays a protumor role during TME progression in the ICS model. We found that this segregation often occurs at a diminished activated CD8^+^ T-cell population and can mark a transition to a stage that shares characteristics (Fig. S5e) with stochastic single-variable growth. We also observed that spatial mixing of activated CD8^+^ T cells and TAMs in the microscale tumor tissues increases the probability of the interactions between these cells leading to increased exhaustion of the CD8^+^ T cells in the TME (Fig. 4c) and subsequent tumor growth. Similar conjectures on the relevance of cellular spatial distributions have been made qualitatively previously (35–41). We were able to deduce such dependencies from the time evolution of patient slides, and our ICS model provided mechanisms that underlie this behavior. In evaluation of the model dynamics, we found fluctuations in cell population play a major role in prediction of patient response to ICI therapy from biopsy slides taken from patients' pretreatment. For some slides (such as 16BL), final cancer cell population counts between simulations can vary so widely due to stochastic fluctuations that they give rise to different predictions of response. For other slides, predictions of response are effectively deterministic. The greater these fluctuations are, the less predictive power the biopsy slides have of patient response.

### Limitations of the approach

We ignored several possibly relevant variables in our ICS model for simplicity and due to difficulties in providing validation against experiments. For example, our ICS model did not include the tumor vasculature—the blood vessels network regulating flow of nutrients and waste products in the TME, which is an important factor for tumor growth (42). Our analysis of the IMC dataset determined endothelial cells (CD31^+^ cells) to be associated with responders to the ICI treatment. Cytokines and chemokines could affect immune cell cytotoxicity, proliferation, exhaustion, and recruitment in the TME. The 3D structure of the tumor that was not explicitly included in the model could affect variations in the TME progression as well. In three dimensions, the exhausted CD8^+^ T cells may be less likely to form fencing structures due to the heterogeneous tumor cell distribution and diffusion. However, we found exhausted CD8^+^ T-cell fencing in the 2D snapshots of 3D tissue samples experimental, suggesting such structures do arise in three dimensions. These factors could affect the rates of the processes in our model, even making them dependent on time. Nevertheless, we expect the mechanisms underlying the phenomena we have identified will play a role in the extended models, although quantitative details can differ.

### Limitations of the data

The datasets that underpin our theoretical modeling have definite limitations arising from the small spatial extent (1 mm × 1 mm) of the TMA and small sample size (30 patients) defined by the patient cohort. The size of the TMA restricts us from unveiling patterns on the scale above 100–200 μm. We only employ techniques to examine cell–cell relationships at smaller length scales (5–50 μm) and how they differ between responders and nonresponder slides. Within each slide, we investigate these spatial relationships by averaging across the hundreds to multiple thousands of resident cells in CyCIF (10) and IMC (5) datasets. Despite the small cohort size, we find differences in densities (*P* < 0.16) and spatial correlations (*P* < 0.05) of specific cell types between responders and nonresponders in the IMC data, such as in the spatial correlation between macrophage/monocytes and activated CD8^+^ T cells.

The lack of experimental information for different circumstances precludes the possibility of modeling them reliably. For example, as none of the patients in the cohort went without ICI therapy, we do not model the TME without ICI therapy applied though we do explore such effects in Fig. S5f and the Sensitivity of patient response section in Supplementary material. Because of the cohort size restriction and lack of information on the treatment regimens, we also did not model any of the three different treatments individually or explicit pharmacodynamics of the ICI drugs. Incorporation of these details could enable the model to provide patient-specific recommendations regarding choice of ICI drugs and their delivery using pretreatment biopsy slides. We plan to include these factors supported by relevant, available experimental data in future iterations of this model.

Despite the limitations of the data, we constructed our model consistent with the results both to clarify the steps in developing models from available data and to obtain useful insights into spatial patterns and temporal evolution from our model simulation. The dataset of Moldoveanu et al. does not identify an exhausted subset of CD8^+^ T cells, making it difficult to investigate the performance of our fencing measure in the prediction of their clinical outcomes. Nevertheless, as shown in the Results section, specific spatial features (e.g. *T*_ex_ fencing) identified from this specific model have been seen in unrelated experiments (10). Furthermore, as more data become available, we hope our approach and results will be useful in unraveling the spatiotemporal dynamics of cancer therapy.

## Materials and methods

### Model training

To train the ICS model parameterized by ***θ***, we maximize a response prediction success score function, score(θ). We use the prediction success frequency *f_i_*(**θ**) per slide *i* as defined in the Approach section to construct the model prediction success score (43) is as follows:


(2) $$\;\mathrm{score}(\theta )=\overset{N}{\underset{i=1}{\prod }}\;\left((1-b)f_{i}(\theta )+b\times \frac{1}{2}\right).$$

Here, N=30 is the number of patient slides and the constant *b* is used to capture the intrinsic limitations of the IMC dataset such as TMA slides not representing the TME at larger length scales (approximately several millimeters to centimeters) well enough (e.g. responder slides devoid of CD8^+^ T cells) or bias in the selection of tissue for TMAs to successfully predict patient response. Therefore, given any single patient slide, our model could at maximum predict patient response outcome with a probability 1−b/2 (<1). The factor ½ arises from the fact that 50% of predictions due to intrinsic uncertainties can coincide with the correct patient response by random chance. We choose the value of the constant b=0.1.

We note that in Moldoveanu et al. (5), the patient response outcomes were determined by irRC where clinicians evaluate response by shrinkage of the tumor size or nondevelopment of new lesions and overall increase/decrease in tumor burden. We capture this determination of response parsimoniously with the fold change of the melanoma cell population in our ICS simulations.

We chose to fit two parameters, the rates of exhaustion of activated CD8^+^ T cells by melanoma and TAMs, because the magnitudes of the rates are not well characterized in the literature, and our preliminary sensitivity analysis found them to strongly affect the prediction success score. All other rates are fixed (Table S1). We will refer to this as the two-parameter or base ICS model. The negative log prediction success score −ln(score(***θ***)) is minimized by varying these two rates. Our model results throughout the manuscript are presented for the parameter values estimated by optimizing score(θ).

### Hypothesis testing

We follow standard hypothesis testing procedures commonly used in resampling statistics (44) to compare the optimized base ICS model to alternate models (hypotheses) of interest. We consider two hypotheses where activated CD8^+^ T cells are not driven to exhaustion by (i) the TAMs or (ii) the melanoma cells. A single-parameter alternate model is constructed by fixing one of these estimated parameters (the corresponding exhaustion rates) in the two-parameter model to zero. The model parameters (***θ*_alt_**) in the alternate single-parameter model are re-estimated using the patient's response data by maximizing score(***θ*_alt_**), as we described in the previous section. Since we only varied these two exhaustion rates in the two-parameter model training, in the alternate model, only the other exhaustion rate was varied during the re-estimation procedure. Next, we set up a nonparametric bootstrap method as described below to generate samples from the two-parameter model and the alternate model for comparing the base and the alternate hypotheses. For the base model, our model training produces a prediction success frequency $f_{i}({\hat{\theta }}_{base})$ for any slide *i*, where ${\hat{\theta }}_{base}$ represents the estimated two parameters in the two-parameter base model. We draw *N* (=30) slides from the original set of *N* (=30) slides randomly with replacement to create a bootstrap sample (indexed by *β*) of slides. Then, we compute the success score function ($[\mathrm{score}({\hat{\theta }}_{base}){]}_{\beta }$) using the prediction success frequencies, $\left\{\;f_{i}({\hat{\theta }}_{base})\right\}$, associated with the slides in the bootstrap sample *β*. The calculation of the success score function is repeated for many bootstrapped samples to generate a set of success scores for the base model. Next, we use the same procedure to generate a set of success scores $\left\{{\left[\mathrm{score}({\hat{\theta }}_{alt})\right]}_{\beta }\right\}$ for the alternate model. We compare the base and the alternate models by computing a test statistic given by the log-difference in the success score (DSS) predictions for base and the alternate model for the bootstrapped samples, i.e. $\mathrm{DS}S_{\beta }=-\ln({\left[\mathrm{score}({\hat{\theta }}_{base})\right]}_{\beta })+\ln\;({\left[\mathrm{score}({\hat{\theta }}_{alt})\right]}_{\beta })=\ln\;\left(\frac{{\left[\mathrm{score}({\hat{\theta }}_{alt})\right]}_{\beta }}{{\left[\mathrm{score}({\hat{\theta }}_{base})\right]}_{\beta }}\right).$

We construct the bootstrap distribution of DSS using multiple bootstrap samples (Figs. 3b and S2). If the one-sided (1−α)×100% CI for DSS does not contain zero, we reject the hypothesis of equality at the α×100% level (i.e. the optimized ICS model provides a better fit to the data than the alternate model, and this improved fit cannot be readily explained by chance alone).

As our framework compares the optimized ICS model to alternate models where one of the two varying model parameters is fixed, the two hypotheses presented are the only hypotheses tested of significant interest (whether or not TAMs or melanoma cells exhaust activated CD8^+^ T cells) which may be tested with our base two-parameter ICS model. Testing whether these rates are any other specific values is of little interest.

Whenever multiple hypotheses are tested simultaneously, *P*-values for each individual hypothesis test should be adjusted. Here, we used a simple Bonferroni adjustment (i.e. each *P*-value was multiplied by the total number of tests performed) to obtain adjusted *P*-values of 14 and 18% for testing hypotheses related to TAM and melanoma cell exhaustion of activated CD8^+^ T cells, respectively. We did not focus on multiple hypotheses testing here, as the adjusted *P*-values are quickly elevated when performing only two simultaneous hypothesis tests and the computational cost is prohibitive.

## Supplementary Material

pgae539_Supplementary_Data

## Data Availability

Data used to generate all figures along with videos of the time evolution of single simulations of slides 06RD and 16BL may be found at DOI: 10.5281/zenodo.10206505. Instructions and code to build and execute ICS model simulations can be found at https://github.com/gdag458/Melanoma_ICS. Moldoveanu et al. make the patient IMC slides produced by their study available at DOI: 10.5281/zenodo.5903179 as noted in their paper (5). The melanoma patient CyCIF data produced by Nirmal et al. (10) is available at https://humantumoratlas.org as noted in their paper.

## Previous Presentation

This manuscript was posted on bioArxiv at DOI: https://doi.org/10.1101/2023.12.06.570410.

## References

[1] Turley SJ, Cremasco V, Astarita JL. 2015. Immunological hallmarks of stromal cells in the tumour microenvironment. Nat Rev Immunol. 15(11):669–682. 26471778 10.1038/nri3902

[2] Steele MM, et al 2023. T cell egress via lymphatic vessels is tuned by antigen encounter and limits tumor control. Nat Immunol. 24(4):664–675. 36849745 10.1038/s41590-023-01443-y PMC10998279

[3] Sharma P, Hu-Lieskovan S, Wargo JA, Ribas A. 2017. Primary, adaptive, and acquired resistance to cancer immunotherapy. Cell. 168(4):707–723. 28187290 10.1016/j.cell.2017.01.017 PMC5391692

[4] Young A, Quandt Z, Bluestone JA. 2018. The balancing act between cancer immunity and autoimmunity in response to immunotherapy. Cancer Immunol Res. 6(12):1445–1452. 30510057 10.1158/2326-6066.CIR-18-0487 PMC6281171

[5] Moldoveanu D, et al 2022. Spatially mapping the immune landscape of melanoma using imaging mass cytometry. Sci Immunol. 7(70):eabi5072. 35363543 10.1126/sciimmunol.abi5072

[6] Karimi E, et al 2023. Single-cell spatial immune landscapes of primary and metastatic brain tumours. Nature. 614(7948):555–563. 36725935 10.1038/s41586-022-05680-3 PMC9931580

[7] Wang XQ, et al 2023. Spatial predictors of immunotherapy response in triple-negative breast cancer. Nature. 621(7980):868–876. 37674077 10.1038/s41586-023-06498-3 PMC10533410

[8] Sorin M, et al 2023. Single-cell spatial landscapes of the lung tumour immune microenvironment. Nature. 614(7948):548–554. 36725934 10.1038/s41586-022-05672-3 PMC9931585

[9] Jackson HW, et al 2020. The single-cell pathology landscape of breast cancer. Nature. 578(7796):615–620. 31959985 10.1038/s41586-019-1876-x

[10] Nirmal AJ, et al 2022. The spatial landscape of progression and immunoediting in primary melanoma at single-cell resolution. Cancer Discov. 12(6):1518–1541. 35404441 10.1158/2159-8290.CD-21-1357 PMC9167783

[11] Lin JR, et al 2018. Highly multiplexed immunofluorescence imaging of human tissues and tumors using t-CyCIF and conventional optical microscopes. Elife. 7:e31657. 29993362 10.7554/eLife.31657 PMC6075866

[12] Berry S, et al 2021. Analysis of multispectral imaging with the AstroPath platform informs efficacy of PD-1 blockade. Science. 372(6547):eaba2609. 34112666 10.1126/science.aba2609 PMC8709533

[13] Li A, et al 2022. Selective targeting of GARP-LTGFβ axis in the tumor microenvironment augments PD-1 blockade via enhancing CD8+ T cell antitumor immunity. J Immunother Cancer. 10(9):e005433. 36096533 10.1136/jitc-2022-005433 PMC9472209

[14] Hammerl D, et al 2021. Spatial immunophenotypes predict response to anti-PD1 treatment and capture distinct paths of T cell evasion in triple negative breast cancer. Nat Commun. 12(1):5668. 34580291 10.1038/s41467-021-25962-0 PMC8476574

[15] Lin JR, et al 2023. Multiplexed 3D atlas of state transitions and immune interaction in colorectal cancer. Cell. 186(2):363–381.e19. 36669472 10.1016/j.cell.2022.12.028 PMC10019067

[16] Schapiro D, et al 2017. histoCAT: analysis of cell phenotypes and interactions in multiplex image cytometry data. Nat Methods. 14(9):873–876. 28783155 10.1038/nmeth.4391 PMC5617107

[17] Chen Z, Soifer I, Hilton H, Keren L, Jojic V. 2020. Modeling multiplexed images with spatial-LDA reveals novel tissue microenvironments. J Comput Biol. 27(8):1204–1218. 32243203 10.1089/cmb.2019.0340 PMC7415889

[18] Wu Z, et al 2022. Graph deep learning for the characterization of tumour microenvironments from spatial protein profiles in tissue specimens. Nat Biomed Eng. 6(12):1435–1448. 36357512 10.1038/s41551-022-00951-w

[19] Milosevic V . 2023. Different approaches to imaging mass cytometry data analysis. Bioinform Adv. 3(1):vbad046. 37092034 10.1093/bioadv/vbad046 PMC10115470

[20] Gong C, et al 2017. A computational multiscale agent-based model for simulating spatio-temporal tumour immune response to PD1 and PDL1 inhibition. J R Soc Interface. 14(134):20170320. 28931635 10.1098/rsif.2017.0320 PMC5636269

[21] Cess CG, Finley SD. 2020. Multi-scale modeling of macrophage-T cell interactions within the tumor microenvironment. PLoS Comput Biol. 16(12):e1008519. 33362239 10.1371/journal.pcbi.1008519 PMC7790427

[22] Konstorum A, Vella AT, Adler AJ, Laubenbacher RC. 2017. Addressing current challenges in cancer immunotherapy with mathematical and computational modelling. J R Soc Interface. 14(131):20170150. 28659410 10.1098/rsif.2017.0150 PMC5493798

[23] Norton KA, Gong C, Jamalian S, Popel AS. 2019. Multiscale agent-based and hybrid modeling of the tumor immune microenvironment. Processes (Basel). 7(1):37. 30701168 10.3390/pr7010037 PMC6349239

[24] Kather JN, et al 2017. In silico modeling of immunotherapy and stroma-targeting therapies in human colorectal cancer. Cancer Res. 77(22):6442–6452. 28923860 10.1158/0008-5472.CAN-17-2006

[25] Mpekris F, et al 2020. Combining microenvironment normalization strategies to improve cancer immunotherapy. Proc Natl Acad Sci U S A. 117(7):3728–3737. 32015113 10.1073/pnas.1919764117 PMC7035612

[26] Hutchinson LG, Grimm O. 2022. Integrating digital pathology and mathematical modelling to predict spatial biomarker dynamics in cancer immunotherapy. NPJ Digit Med. 5(1):92. 35821064 10.1038/s41746-022-00636-3 PMC9276679

[27] Cess CG, Finley SD. 2023. Calibrating agent-based models to tumor images using representation learning. PLoS Comput Biol. 19(4):e1011070. 37083821 10.1371/journal.pcbi.1011070 PMC10156003

[28] Wolchok JD, et al 2009. Guidelines for the evaluation of immune therapy activity in solid tumors: immune-related response criteria. Clin Cancer Res. 15(23):7412–7420. 19934295 10.1158/1078-0432.CCR-09-1624

[29] Chaikin PM, Lubensky TC. 1995. Principles of condensed matter physics. Cambridge: Cambridge University Press.

[30] Seager RJ, Hajal C, Spill F, Kamm RD, Zaman MH. 2017. Dynamic interplay between tumour, stroma and immune system can drive or prevent tumour progression. Converg Sci Phys Oncol. 3:034002. 30079253 10.1088/2057-1739/aa7e86 PMC6070160

[31] Carlino MS, Larkin J, Long GV. 2021. Immune checkpoint inhibitors in melanoma. Lancet. 398(10304):1002–1014. 34509219 10.1016/S0140-6736(21)01206-X

[32] Sabbatino F, Liguori L, Pepe S, Ferrone S. 2022. Immune checkpoint inhibitors for the treatment of melanoma. Expert Opin Biol Ther. 22(5):563–576. 35130816 10.1080/14712598.2022.2038132 PMC9038682

[33] Rodriguez-Murillo L, et al 2010. Novel loci interacting epistatically with bone morphogenetic protein receptor 2 cause familial pulmonary arterial hypertension. J Heart Lung Transplant. 29(2):174–180. 19864167 10.1016/j.healun.2009.08.022 PMC2815041

[34] Pinsky MA, Karlin S. 6—Continuous time Markov chains. 2011. In: Pinsky MA, Karlin S, editors. An introduction to stochastic modeling. 4th ed. Boston: Academic Press. p. 277–346.

[35] Kakavand H, et al 2015. Tumor PD-L1 expression, immune cell correlates and PD-1+ lymphocytes in sentinel lymph node melanoma metastases. Mod Pathol. 28(12):1535–1544. 26403784 10.1038/modpathol.2015.110

[36] Saldanha G, Flatman K, Teo KW, Bamford M. 2017. A novel numerical scoring system for melanoma tumor-infiltrating lymphocytes has better prognostic value than standard scoring. Am J Surg Pathol. 41(7):906–914. 28368925 10.1097/PAS.0000000000000848 PMC6171741

[37] Keun Park C, Kyum Kim S. 2017. Clinicopathological significance of intratumoral and peritumoral lymphocytes and lymphocyte score based on the histologic subtypes of cutaneous melanoma. Oncotarget. 8(9):14759–14769. 28107203 10.18632/oncotarget.14736 PMC5362441

[38] Obeid JM, et al 2016. PD-L1, PD-L2 and PD-1 expression in metastatic melanoma: correlation with tumor-infiltrating immune cells and clinical outcome. Oncoimmunology. 5(11):e1235107. 27999753 10.1080/2162402X.2016.1235107 PMC5139635

[39] Weiss SA, et al 2016. Immunologic heterogeneity of tumor-infiltrating lymphocyte composition in primary melanoma. Hum Pathol. 57:116–125. 27473267 10.1016/j.humpath.2016.07.008 PMC5706446

[40] Fortes C, et al 2015. Tumor-infiltrating lymphocytes predict cutaneous melanoma survival. Melanoma Res. 25(4):306–311. 25933208 10.1097/CMR.0000000000000164

[41] Song H, Wu Y, Ren G, Guo W, Wang L. 2015. Prognostic factors of oral mucosal melanoma: histopathological analysis in a retrospective cohort of 82 cases. Histopathology. 67(4):548–556. 25809697 10.1111/his.12692

[42] Franses JW, Baker AB, Chitalia VC, Edelman ER. 2011. Stromal endothelial cells directly influence cancer progression. Sci Transl Med. 3(66):66ra5. 10.1126/scitranslmed.3001542 PMC3076139 21248315

[43] van der Vaart AW. 2000. Asymptotic statistics. Cambridge: Cambridge University Press.

[44] Hastie T, Tibshirani R, Friedman J. Model inference and averaging. 2009. In: Hastie T, Tibshirani R, Friedman J, editors. The elements of statistical learning: data mining, inference, and prediction. New York, NY: Springer New York. p. 261–294.

