# Supplementary material for: Spatial organization and stochastic fluctuations of immune cells impact clinical responsiveness to immunotherapy in melanoma patients
Source: PNAS Nexus. 2024 Nov 26;3(12):pgae539. doi: 10.1093/pnasnexus/pgae539 (PMC11642613; doi:10.1093/pnasnexus/pgae539)
Supplement: pgae539_Supplementary_Data [file pgae539_supplementary_data.zip › PNASNEXUS-PNASNEXUS-2024-00741-TR-s03.pdf]

**a**

| | |
|---|---|
| Melanoma | 0.951 |
| all CD8+ | 0.197 |
| Naïve CD8+ | 0.508 |
| Activated CD8+ | 0.163 |
| All CD4+ | 0.602 |
| Naïve CD4+ | 0.199 |
| Activated CD4+ | 0.914 |
| Treg | 0.4 |
| Macrophage/Monocytes | 0.64 |
| CD31+ | 0.669 |
| B Cells | 0.251 |

**b**

| | Melanoma Cells | all CD8+ T Cells | Naïve CD8+ T Cells | Activated CD8+ T Cells | All CD4+ T Cells | Naïve CD4+ T Cells | Activated CD4+ T Cells | Treg Cells | Macrophage/Monocytes | CD31+ Cells | B Cells |
|---|---|---|---|---|---|---|---|---|---|---|---|
| Melanoma Cells | 0.567 | 0.641 | 0.634 | 0.565 | 0.551 | 0.186 | 0.915 | 0.273 | 0.363 | 0.57 | 0.206 |
| all CD8+ T Cells | 0.061 | 0.227 | 0.855 | 0.169 | 0.736 | 0.41 | 0.877 | 0.531 | 0.042 | 0.638 | 0.313 |
| Naïve CD8+ T Cells | 0.275 | 0.766 | 0.394 | 0.928 | 0.628 | 0.49 | 0.776 | 0.501 | 0.466 | 0.45 | 0.347 |
| Activated CD8+ T Cells | 0.149 | 0.192 | 0.611 | 0.186 | 0.543 | 0.599 | 0.487 | 0.572 | 0.016 | 0.398 | 0.321 |
| All CD4+ T Cells | 0.373 | 0.908 | 0.374 | 0.876 | 0.728 | 0.385 | 0.865 | 0.315 | 0.235 | 0.049 | 0.369 |
| Naïve CD4+ T Cells | 0.412 | 0.134 | 0.634 | 0.191 | 0.527 | 0.133 | 0.504 | 0.238 | 0.819 | 0.202 | 0.343 |
| Activated CD4+ T Cells | 0.469 | 0.906 | 0.369 | 0.898 | 0.777 | 0.387 | 0.903 | 0.38 | 0.237 | 0.25 | 0.378 |
| Treg Cells | 0.29 | 0.445 | 0.108 | 0.25 | 0.367 | 0.184 | 0.3 | 0.658 | 0.378 | 0.574 | 0.199 |
| Macrophage/Monocytes | 0.106 | 0.004 | 0.96 | 0.002 | 0.061 | 0.434 | 0.083 | 0.529 | 0.026 | 0.224 | 0.216 |
| CD31+ Cells | 0.744 | 0.369 | 0.811 | 0.327 | 0.259 | 0.798 | 0.222 | 0.674 | 0.158 | 0.713 | 0.192 |
| B Cells | 0.154 | 0.176 | 0.97 | 0.171 | 0.72 | 0.693 | 0.741 | 0.344 | 0.241 | 0.406 | 1 |

**Fig S1. Significant variations of spatial relationships between responders and non-responders are yielded from the data. (a)** Table of p-values corresponding to the average density of each species varying between responder slides and non-responder slides. The average activated CD8+ T cell densities are separated with the most confidence with a p-value of 0.16. Responder slides have more activated CD8+ T cells on average. **(b)** Table of p-values corresponding to the average spatial correlations at 10.5 $\mu m$ varying between responder and non-responder slides for all permutations of cell type. Values below 0.05 are starred.
