# Supplementary material for: Spatial organization and stochastic fluctuations of immune cells impact clinical responsiveness to immunotherapy in melanoma patients
Source: PNAS Nexus. 2024 Nov 26;3(12):pgae539. doi: 10.1093/pnasnexus/pgae539 (PMC11642613; doi:10.1093/pnasnexus/pgae539)
Supplement: pgae539_Supplementary_Data [file pgae539_supplementary_data.zip › PNASNEXUS-PNASNEXUS-2024-00741-TR-s05.pdf]

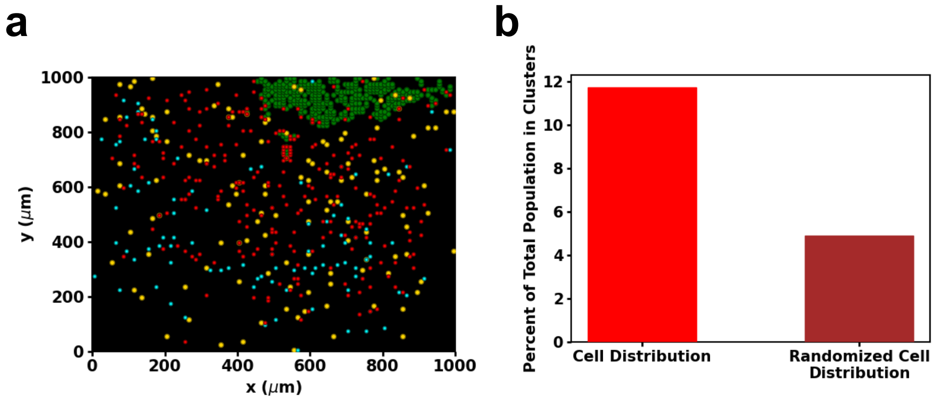

Fig. S3. Exhausted CD8+ T cell fencing exhibited in slide 16BL time-evolution. (a) Snapshot of the simulated TME of 16BL at 144 hours exhibiting fencing clusters. **(b)** The percentage of exhausted CD8+ T cells in fencing clusters at 144 hours in a simulation of slide 16BL compared to that expected from a randomly permuted distribution of exhausted CD8+ T cells.
