# Supplementary material for: Spatial organization and stochastic fluctuations of immune cells impact clinical responsiveness to immunotherapy in melanoma patients
Source: PNAS Nexus. 2024 Nov 26;3(12):pgae539. doi: 10.1093/pnasnexus/pgae539 (PMC11642613; doi:10.1093/pnasnexus/pgae539)
Supplement: pgae539_Supplementary_Data [file pgae539_supplementary_data.zip › PNASNEXUS-PNASNEXUS-2024-00741-TR-s06.pdf]

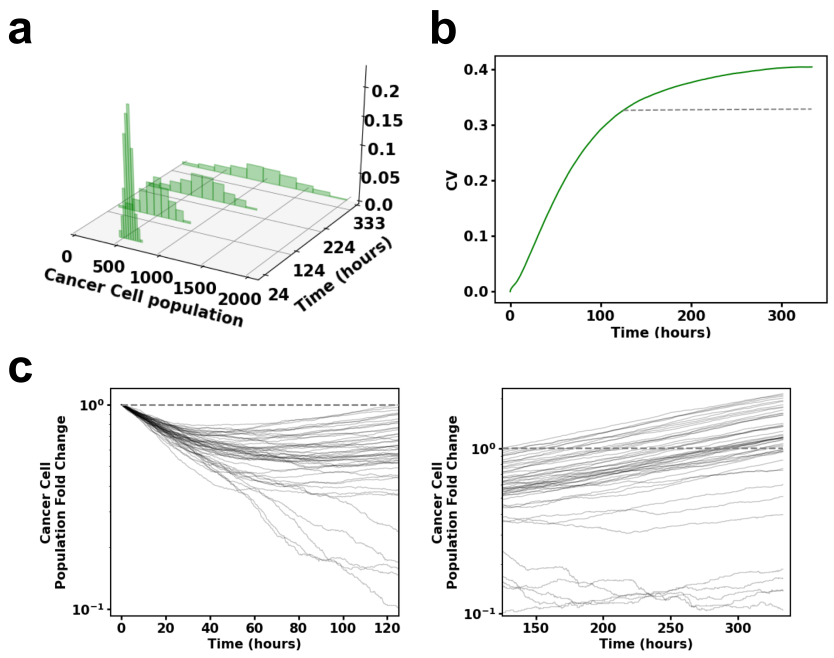

Fig. S4. Characterizing stochasticity in melanoma cell population trajectories corresponding to slide 16BL further. (a) Cancer cell population distribution obtained from the same 1000 samples as in (a) at increasing times showing how the cancer cell population distribution spreads over time. **(b)** The coefficient of variation (σ/μ) for 1000 simulations of slide 16BL (green) with the fitted Yule coefficient of variation (grey dashed line) from 125 hours. **(c)** 50 melanoma fold-change trajectories from simulations of 16BL with the trained model plotted from initial time to 125 hours (left) and then from 125 hours to final time (right). We see that early on (around 24 hours to 80 hours), trajectories intersect often whereas at later times (past 125 hours) the trajectories largely remain separated.
