# Supplementary material for: Spatial organization and stochastic fluctuations of immune cells impact clinical responsiveness to immunotherapy in melanoma patients
Source: PNAS Nexus. 2024 Nov 26;3(12):pgae539. doi: 10.1093/pnasnexus/pgae539 (PMC11642613; doi:10.1093/pnasnexus/pgae539)
Supplement: pgae539_Supplementary_Data [file pgae539_supplementary_data.zip › PNASNEXUS-PNASNEXUS-2024-00741-TR-s07.pdf]

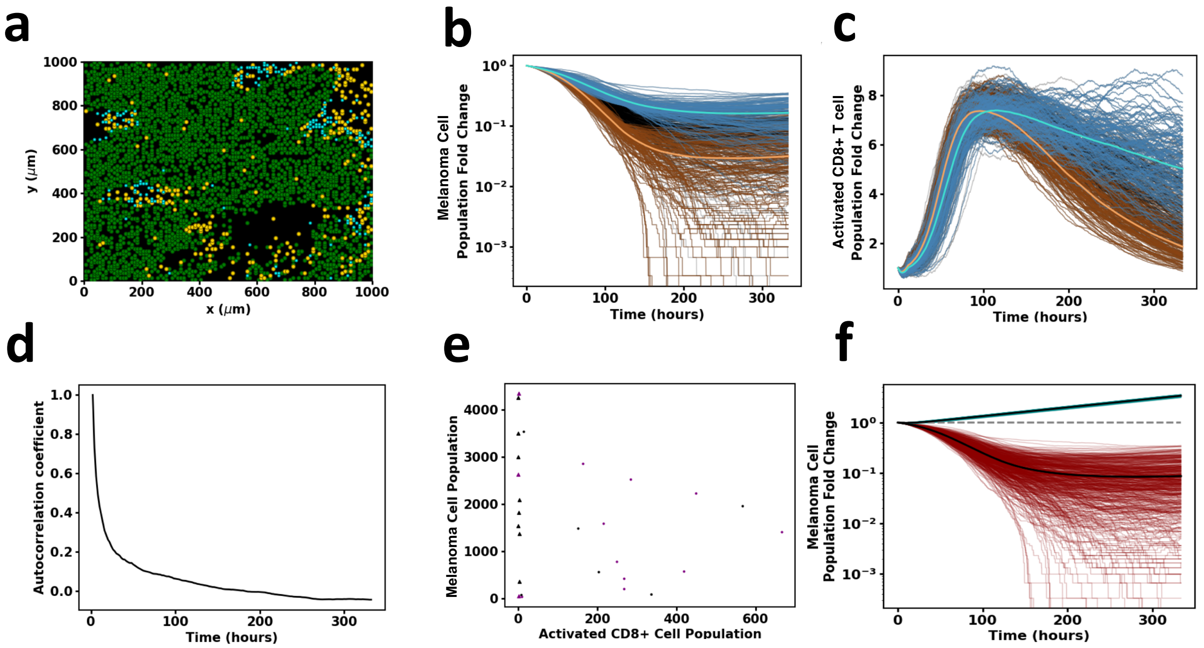

Fig. S5. Stochasticity in ICS-simulated slide 06RD displays the trajectory-mixing stage. (a) Initial cell spatial distribution for slide 06RD. **(b)** Melanoma cell (log-linear plot) and **(c)** activated CD8+ T cell population (linear-linear plot) trajectories plotted for 1000 samples of slide 06RD. Blue (brown) trajectories are those which compose the top (bottom) 25% melanoma cell populations across all samples at 125 hours. The lighter blue (brown) trajectory corresponds to the average trajectory of all of the blue (brown) trajectories. The black trajectories represent the other 50% of samples. All samples begin with identical initial conditions set by the patient slide data 06RD. Notice how sample trajectories mix until late times unlike in simulations of slide 16BL at late times. 06RD kinetics are also characterized by maintenance of a larger activated CD8+ T cell population until late times. **(d)** The autocorrelation of cancer cell population from 2 hours to final time for slide 06RD. The autocorrelation quickly drops showing that there is low predictive power of future state given initial cancer cell population for this slide. **(e)** Average cancer cell population across 300 samples for every slide at 25 hours plotted against the average activated CD8+ T cell population over the same samples at 25 hours. Purple (black) markers delineate responders (non-responders) and triangles (circles) mark slides which have (not) transitioned into the simple growth stage by 25 hours. Those slides which start as simple growth processes can be separated out by average activated CD8+ T cell population. **(f)** Melanoma cell population trajectories through time from 500 simulations of patient slide 06RD with the base model where each dark red line shows a simulation run of the stochastic ICS model and the average is shown with a solid black line. Individual trajectories of the melanoma cell population for the modified base model where specific rate parameters are altered to represent the ICI drug treatment-naïve case are shown in teal lines with the solid black line depicting the average of the trajectories. We increased the rates of exhaustion of activated CD8+ T cells (×1.5), decreased the rate of lysis of melanoma cells (×0.666), and decreased the rate of proliferation of activated CD8+ T cells (×0.666) from their respective values in the base model to represent the treatment-naïve case. The simulations show the responder slide 06RD is turned into a non-responder in the absence of the ICI drug therapy.
