# Supplementary material for: Spatial organization and stochastic fluctuations of immune cells impact clinical responsiveness to immunotherapy in melanoma patients
Source: PNAS Nexus. 2024 Nov 26;3(12):pgae539. doi: 10.1093/pnasnexus/pgae539 (PMC11642613; doi:10.1093/pnasnexus/pgae539)
Supplement: pgae539_Supplementary_Data [file pgae539_supplementary_data.zip › PNASNEXUS-PNASNEXUS-2024-00741-TR-s08.pdf]

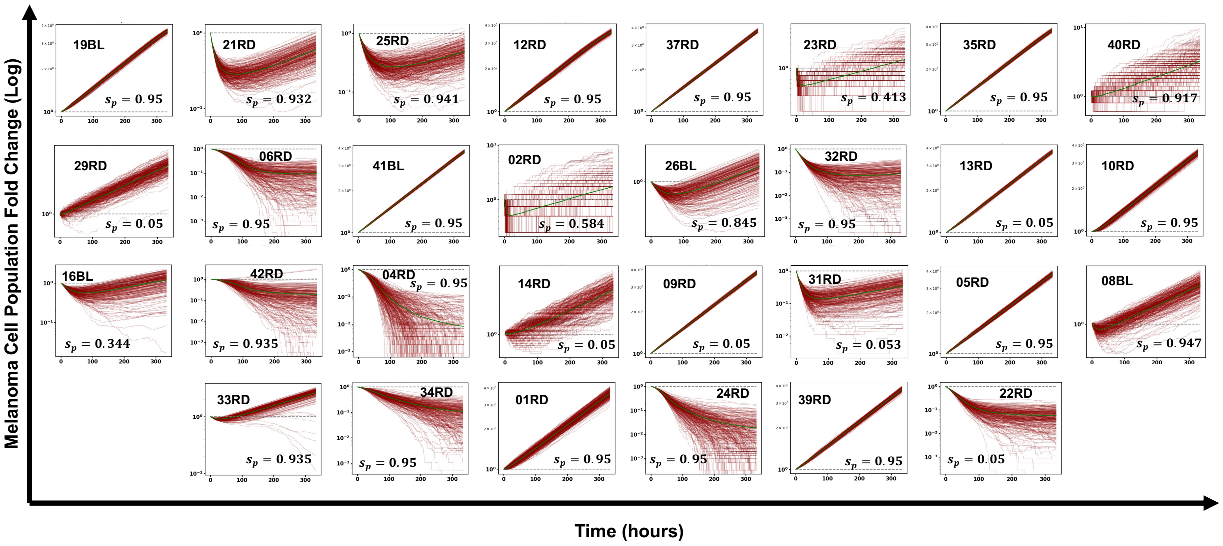

Fig. S6. All slide melanoma cell population trajectories. Log-linear plots of melanoma cell population trajectories for 300 samples simulated with optimal parameters and initialized by each IMC slide. The average trajectory for each slide is plotted in green. We include the prediction success for each slide, $s_p = (1-b)f_i(\boldsymbol{\theta}_{\mathbf{opt}}) + b \times \frac{1}{2}$, with b and $f_i(\boldsymbol{\theta}_{\mathbf{opt}})$ defined as in the Model Training section of Materials and Methods. Note that certain slides have initial conditions that are not conducive to prediction such as responder slide 09RD which has no initial CD8+ T cells. For such slides, poor results are expected.
