# Supplementary material for: Spatial organization and stochastic fluctuations of immune cells impact clinical responsiveness to immunotherapy in melanoma patients
Source: PNAS Nexus. 2024 Nov 26;3(12):pgae539. doi: 10.1093/pnasnexus/pgae539 (PMC11642613; doi:10.1093/pnasnexus/pgae539)
Supplement: pgae539_Supplementary_Data [file pgae539_supplementary_data.zip › PNASNEXUS-PNASNEXUS-2024-00741-TR-s09.docx]

**Table S1. Processes, rules, and parameters used in the ICS mode**l. All parameters are held fixed through all simulations except for the two rates which are estimated through the training process: the exhaustion rates of activated CD8+ T cells by both melanoma cells ($b_{C}$) and TAMs ($b_{M}$).

| **Processes** | **Rules** | **Rate** | **Notes** |
| --- | --- | --- | --- |
| Melanoma Cell Proliferation | $C\underset{\to}{k_{C}}C+C$ | $k_{C}=0.096 d^{-1}$  (21) | The ability of melanoma cells to push aside CD8+ T cells has been similarly executed in other computational models (22). Details are described in the Materials and Methods section. The rate was measured in in vitro assays using primary melanoma cells obtained from biopsy samples (21). |
| Activated CD8+ T cell recruitment | $\phi\underset{\to}{r}T_{C}$  Equation (5) | ${r=0.188 hr}^{-1}$ | CD8+ T cells are recruited to the TME in an activated state (1, 2, 23-25). The rate was fixed such that activated CD8+ T recruitment can recuperate the cell population number when small but has reduced effectiveness at larger populations. More details in the Approach section. |
| Activated CD8+ T cell proliferation | $T_{C}\underset{\to}{k_{pro}}T_{C}+T_{C}$  Equation (4) | ${k_{\mathrm{pro}}=0.090 hr}^{-1}$  (26, 27) | Activated CD8+ T cells proliferate in the TME unlike exhausted CD8+ T cells (2, 3, 14). The rate is within an order of magnitude of the proliferation rate ($0.222 hr^{-1}$) observed in vivo in CD8^+^ T cells obtained from mouse draining lymph nodes (26). |
| CD8+ T cell population limit | Total CD8+ T cell population may not exceed $N_{CC}$  Equations (4, 5) | $N_{CC}=3000$ CD8+ T cells | The carrying capacity for all CD8+ T cells is set to roughly four times the maximum CD8+ T cell population found in the IMC slides (20). |
| Influence of dead melanoma cells on activated CD8+ T cell proliferation and recruitment | Michaelis constant, $D_{\frac{1}{2}}$  Equations (4, 5) | $D_{\frac{1}{2}}=200$ lysed melanoma cells | A similar Michaelis-Menten dependence on recently lysed melanoma cells has been used before (22). The value of 200 lysed melanoma cells was chosen, upon model testing, such that activated CD8+ T cell recruitment and proliferation maintain dependence on neoantigen load and are not commonly zero. More details in the Materials and Methods section. |
| Span of immune system melanoma cell lysis memory | System memory span of number of lysed melanoma cells influencing activated CD8+ T cell dynamics More details in the Methods section. $T_{m}$ | $T_{m}=12 hr$ | Neoantigens produced by lysed melanoma cells are captured by APCs then presented to activated CD8+ T cells in the TME, or trafficked to the tumor draining lymph nodes and activated CD8+ T cells which enter the TME via circulation (25). Antigens are presented on the surface of cells in as little as 30 minutes (28) as peptides bound to MHC-I. More details in the Materials and Methods section. |
| Activated CD8+ T Cell Lysing Melanoma Cell | $T_{C}+C\underset{\to}{l}T_{C}$ | $l=0.360 {hr}^{-1}$  (29) | The lag time between OVA specific CD8+ T cells binding to a target cell until target cell lysis was measured with time-lapse microscopy (29) which is to be within the range $0.303-3.33 hr^{-1}$. We choose a value close to the lower part of the range. |
| CD8+ T Cell motility | Cells diffuse into neighboring chambers with enough space, $D_{mot}$ | $D_{mot}=2.375 \mu m^{2}/min$  (11, 22) | T cell movement observed using 2-photon microscopy in the mouse melanoma TME resemble a random walk and move $1-2\mu m/min$(11). We computed the diffusion constant relating the average distance traversed $\Delta x$ in a time interval $\tau$ as $D_{mot}=\Delta x^{2}/2\tau$. The diffusion constant used also matches previous computational models (30). More details in the Materials and Methods section. |
| CD8+ T cell adhering to melanoma cell when in contact | Reduced diffusion effectively models CD8+ T cells adhering to melanoma cells, $D_{ad}$ | $D_{ad}=0.071 \mu m^{2}/min$ | CD8+ T cells adhere to target melanoma cells (31, 32). The rate is fixed such that the cells remain in contact long enough to interact via cytotoxic action or exhaustion interactions. More details in the Materials and Methods section. |
| Activated CD8+ T Cell Exhaustion by Melanoma Cell | $T_{C}+C\underset{\to}{b_{C}}T_{E}+C$ | $b_{C}=0.064\pm0.02 hr^{-1}$ | Rate estimated through model training. |
| Activated CD8+ T Cell Exhaustion by TAM | $T_{C}+M\underset{\to}{b_{M}}T_{E}+M$ | $b_{M}=0.232 \pm0.04 hr^{-1}$ | Rate estimated through model training. |
| Zero TAM Exhaustion Alternate Model Activated CD8+ T Cell Exhaustion by Melanoma Cell | $T_{C}+C\underset{\to}{b_{C}}T_{E}+C$ | $b_{C}=0.063 hr^{-1}$ | Rate estimated through model training. |
| Zero Melanoma Cell Exhaustion Alternate Model Activated CD8+ T Cell Exhaustion by Melanoma Cell | $T_{C}+C\underset{\to}{b_{C}}T_{E}+C$ | $b_{M}=0.301 hr^{-1}$ | Rate estimated through model training. |
| Exhausted CD8+ T Cell Death | $T_{E}\underset{\to}{\gamma_{E}}\phi$ | $\gamma_{E}=2 {wk}^{-1}$  (33) | In one study (33), the activated CD8+ T cell death rate was approximated for CD8+ T cells in LCMV-infected mice. We assume the calculated values for activated CD8+ T cell death are relevant to exhausted CD8+ T cells in the TME. The range of values calculated is $0.13-2.8 wk^{-1}$. |
| TAM Recruiting | $\phi\underset{\to}{r_{M}}M$ | ${r_{M}=0.833 hr}^{-1}$  (34) | TAMs are recruited into the TME (35). The rate is close to what has been used in previous models of the TME ($0.36 hr^{-1}$) (34). More details in the Approach section. |
| TAM Death | $M\underset{\to}{\gamma_{M}}\phi$ | ${\gamma_{M}=0.286 d}^{-1}$  (36, 37) | The death rate is that for monocytes in a human (36). Human cells were labeled in vivo and tracked over time then mathematical models were used to yield lifespans. |
| TAM Motility | TAMs diffuse into neighboring chambers with enough space, $D_{M}$ | $D_{M}=0.594 \mu m^{2}/min$  (38-40) | The diffusion constant for TAM diffusion roughly matches the time scale of motility of mouse macrophages in transwell migration (38) and TAXIScan (39) assays which produce a range of speeds from roughly $0.55-1.0 \mu/min$ (38). |
