# Supplementary material for: Spatial organization and stochastic fluctuations of immune cells impact clinical responsiveness to immunotherapy in melanoma patients
Source: PNAS Nexus. 2024 Nov 26;3(12):pgae539. doi: 10.1093/pnasnexus/pgae539 (PMC11642613; doi:10.1093/pnasnexus/pgae539)
Supplement: pgae539_Supplementary_Data [file pgae539_supplementary_data.zip › PNASNEXUS-PNASNEXUS-2024-00741-TR-s10.docx]

**Table S2. Sensitivity analysis.** The optimal negative log prediction success score (21.1, predicting 78% of slides correctly) was calculated as discussed in Model Training of the Materials and Methods section. To find how this score deviates from the optimal score with parameter perturbations, we multiplied each parameter (indexed by $i$ listed in the table) independently by factors of 0.1 (-) or 10 (+) and then calculated the associated prediction success scores ($s_{i,\pm}$) with that parameter altered. The variation from optimal score ($f_{i,\pm}$) can be calculated using the score found at optimal parameters $s_{opt}$, $f_{i,\pm}=\frac{s_{i,\pm}-s_{opt}}{s_{opt}}$. The variation corresponding to the melanoma cell proliferation rate is equivalent to predicting only 52% of the slides correctly. With both $f_{i,+}$ and $f_{i,-}$ calculated, the larger is the maximal variation from the optimal score. We report the maximal variation from the optimal score from varying each parameter here.

| **Parameter** | **Maximal variation from optimal score** |
| --- | --- |
| Melanoma cell proliferation | 1.01 |
| Melanoma cell exhaustion of CD8+ T cells | 1.00 |
| CD8+ T cell lysing of melanoma cells | 0.99 |
| CD8+ T cell proliferation | 0.98 |
| TAM exhaustion of CD8+ T cells | 0.81 |
| CD8+ T cell diffusion | 0.66 |
| TAM recruitment | 0.44 |
| CD8+ T cell recruitment | 0.12 |
| TAM diffusion | 0.07 |
