# Supplementary material for: Spatial organization and stochastic fluctuations of immune cells impact clinical responsiveness to immunotherapy in melanoma patients
Source: PNAS Nexus. 2024 Nov 26;3(12):pgae539. doi: 10.1093/pnasnexus/pgae539 (PMC11642613; doi:10.1093/pnasnexus/pgae539)
Supplement: pgae539_Supplementary_Data [file pgae539_supplementary_data.zip › PNASNEXUS-PNASNEXUS-2024-00741-TR-s11.docx]

**Table S3.** **Model Prediction Success with Initial Spatial Perturbations.** For each patient slide, we report the weighted fraction of trajectories (200 trajectories each slide) which correctly predict each patient response with the optimized base ICS model ($\left( 1-b \right)f_{i}\left( \boldsymbol{\theta} \right)+b\times\frac{1}{2}$) given different initial conditions: a) patient slides b) patient slides with randomized TAM distributions c) patient slides with randomized activated CD8+ T cell distributions. Values which differ from the patient slide prediction successes by more than 60% and their corresponding slides are boldened.

| **Slides** | **Optimized Base ICS Model Prediction Success** | **Initially Randomized TAMs ICS Model Prediction Success** | **Initially Randomized Activated CD8s ICS Model Prediction Success** |
| --- | --- | --- | --- |
| 12RD | 0.95 | 0.95 | 0.95 |
| 37RD | 0.95 | 0.95 | 0.95 |
| 23RD | 0.413 | 0.401 | 0.5945 |
| 35RD | 0.95 | 0.95 | 0.95 |
| 40RD | 0.917 | 0.932 | 0.905 |
| 29RD | 0.05 | 0.05 | 0.05 |
| 06RD | 0.95 | 0.95 | 0.95 |
| 41BL | 0.95 | 0.95 | 0.95 |
| 02RD | 0.584 | 0.635 | 0.68 |
| **26BL** | 0.845 | 0.95 | **0.05** |
| 32RD | 0.95 | 0.95 | 0.95 |
| 13RD | 0.05 | 0.05 | 0.05 |
| 10RD | 0.95 | 0.95 | 0.9455 |
| **16BL** | 0.344 | **0.95** | **0.95** |
| 42RD | 0.935 | 0.95 | 0.95 |
| 04RD | 0.95 | 0.95 | 0.95 |
| 14RD | 0.05 | 0.05 | 0.059 |
| 09RD | 0.05 | 0.05 | 0.05 |
| 31RD | 0.053 | 0.092 | 0.0635 |
| 05RD | 0.95 | 0.95 | 0.95 |
| 08BL | 0.947 | 0.926 | 0.95 |
| **33RD** | 0.935 | 0.95 | **0.05** |
| 34RD | 0.95 | 0.95 | 0.95 |
| 01RD | 0.95 | 0.95 | 0.95 |
| 24RD | 0.95 | 0.95 | 0.95 |
| 39RD | 0.95 | 0.95 | 0.95 |
| 22RD | 0.05 | 0.05 | 0.05 |
| 25RD | 0.941 | 0.95 | 0.95 |
| **21RD** | 0.932 | **0.098** | 0.95 |
| 19BL | 0.95 | 0.95 | 0.95 |
