# Supplementary material for: Spatial organization and stochastic fluctuations of immune cells impact clinical responsiveness to immunotherapy in melanoma patients
Source: PNAS Nexus. 2024 Nov 26;3(12):pgae539. doi: 10.1093/pnasnexus/pgae539 (PMC11642613; doi:10.1093/pnasnexus/pgae539)
Supplement: pgae539_Supplementary_Data [file pgae539_supplementary_data.zip › PNASNEXUS-PNASNEXUS-2024-00741-TR-s12.docx]

**Table S4.** **Exhausted CD8+ T cell Fencing Occurrence.** Snapshots of the simulated TME were collected from multiple time points for each slide (one simulation each slide). The number increase of exhausted CD8+ T cells participating in fencing clusters from the snapshots with randomly permuted exhausted CD8+ T cells to those found in the unaltered snapshot was recorded for each timepoint of the simulation of each slide. This difference was then divided by the total number of exhausted CD8+ T cells at that time point in that slide. This fraction increase in T_ex_ fencing clusters informs if T_ex_ fencing is occurring more than expected from a randomized distribution of exhausted CD8+ T cells. The maximum fraction increase in T_ex_ fencing clusters across all time points for each slide is reported above as the fencing metric.

| **Slides** | **Fencing Metric** |
| --- | --- |
| 12RD | 0 |
| 37RD | 0 |
| 23RD | 0.047 |
| 35RD | 0 |
| 40RD | 0 |
| 29RD | 0 |
| 06RD | 0.444 |
| 41BL | 0.75 |
| 02RD | 0.035 |
| 26BL | 0.375 |
| 32RD | 0.325 |
| 13RD | 0.5 |
| 10RD | 0.288 |
| 16BL | 0.235 |
| 42RD | 0.27 |
| 04RD | 0.258 |
| 14RD | 0 |
| 09RD | 0 |
| 31RD | 0.15 |
| 05RD | 0 |
| 08BL | 0.273 |
| 33RD | 0.214 |
| 34RD | 0.271 |
| 01RD | 0 |
| 24RD | 0.174 |
| 39RD | 0 |
| 22RD | 0.279 |
| 25RD | 0.302 |
| 21RD | 0.33 |
| 19BL | 0.5 |
