# Supplementary material for: Spatial organization and stochastic fluctuations of immune cells impact clinical responsiveness to immunotherapy in melanoma patients
Source: PNAS Nexus. 2024 Nov 26;3(12):pgae539. doi: 10.1093/pnasnexus/pgae539 (PMC11642613; doi:10.1093/pnasnexus/pgae539)
Supplement: pgae539_Supplementary_Data [file pgae539_supplementary_data.zip › PNASNEXUS-PNASNEXUS-2024-00741-TR-s14.docx]

**
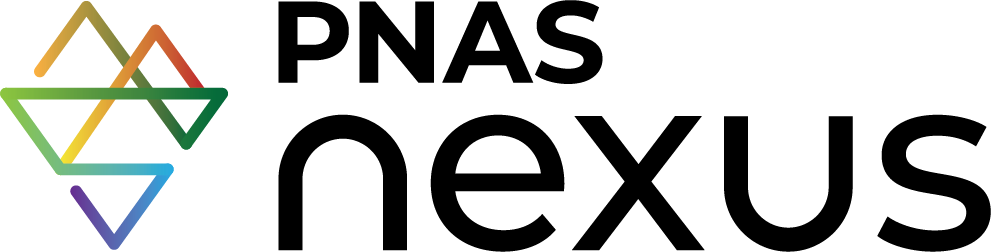
**

**Supporting Information for**

Spatial organization and stochastic fluctuations of immune cells impact clinical responsiveness to immunotherapy in melanoma patients

Giuseppe Giuliani, William Stewart, Zihai Li, Ciriyam Jayaprakash, Jayajit Das

Jayajit Das

Email: jayajit@gmail.com

**This docx file includes:**

Legends for Movies S1 and S2

**Other supporting materials for this manuscript include the following:**

Movies S1 to S2

*Analysis of IMC datasets*: These calculations include cell densities and quantifying spatial organization of different cell types with tools such as the two-point correlation.

Density: The density (σ) of a particular cell type is given by $\sigma=\frac{N_{Tot}}{A}$, where $N_{Tot}$ is the total number of cells of that type and A is the area of the cell tissue in that slide. The area A is calculated by partitioning the slide into a square lattice with lattice constant $a=30 \mu m$ and finding all lattice squares which contain cells. These occupied squares comprise the tissue region of the slide and constitute area of the cell tissue.

Spatial Correlation: We computed the spatial correlation, e.g., between macrophages/monocytes and CD8+ T cells, for a slide in the following way. For each macrophage/monocyte (indexed by i) in the slide, we compute the density of CD8+ T cells in an annular region of radius r and thickness $\delta(=3 \mu m$) centered around the macrophage/monocyte. Let $n_{i\left( r-\delta/2, r+\delta/2 \right)}$ be the number of CD8^+^ T cells in the annular region with area A_annulus_=2πrδ; the density σ*_i_*(*r*) of CD8+ T cells in the annular region surrounding the i^th^ macrophage/monocyte is

$$\sigma_{i}\left( r \right)= \frac{n_{i,\left( r-\frac{\delta}{2}, r+\frac{\delta}{2} \right)}}{A_{Annulus}} .$$

( 2 )

The average density of CD8+ T cells, $\sigma_{CD8+}=\frac{N_{CD8+}}{A}$ is calculated as discussed earlier.

The spatial correlation function is then given by,

$$C\left( r \right)=\frac{1}{N_{macro}}\sum_{i} \sigma_{i}(r) - \sigma_{\mathrm{CD}8^{+} .}$$

( 3 )

This is then scaled by the average density of CD8+ T cells across all slides $\sigma_{cohort, CD8+}$. This results in a unitless metric that determines if the clustering of CD8+ T cells around macrophages/monocytes is above or below what is expected from a random distribution and scaled by the average CD8+ T cell density in a slide (Fig. S1b). The scaled correlation function is plotted as a function of r in Figure 2b.

Model Simulation

The ICS model is a kinetic Monte Carlo simulation implemented on a 100x100 square lattice with spacing 10 $\mu m$ representing the 1mm$\times$1mm TMAs taken from each patient. We customized SPPARKS Kinetic Monte Carlo Simulator (distributed at spparks.github.io) code to build our model. The references supporting model rules and parameter values are shown in Table 1. The physical extent of the cells constrains the number of cells in each chamber that corresponds to a 10 $\mu m \times10 \mu m$ square. CD8+ T cells (active or exhausted) occupy a fourth of a chamber while melanoma cells and macrophages each fill half a chamber. These occupation rules reflect the limits of occupation in the original IMC slides. Two cells are considered in contact if they are in the same chamber or in adjacent chambers that share an edge.

The initial state of the system is obtained from the patient’s TMA by discretizing the cell positions in the image. Over time, cell positions and numbers change: First, melanoma cells proliferate (rate $k_{C}$) with the location of the daughter depending on the availability of space in progressively larger neighboring regions. The new cell is placed in the same chamber as the proliferating cell if space is available, if not then the surrounding 8 chambers, next the adjacent layer of 16 chambers and finally the third layer of 24 chambers are checked for available space. When space is not available in a layer, we allow CD8+ T cells in any of the chambers in that layer to be expelled into available space, and check if proliferation can proceed. If space cannot be found in the 49-cell neighborhood, even by expelling CD8+ T cells, then proliferation does not occur. When multiple chambers are available in a layer, one is chosen randomly.

The CD8+ T cells are relocated by iteratively searching their surrounding layers as in melanoma proliferation until a position to place them is found (with no limit). Once open positions for the CD8+ T cells are found, they are placed randomly. This maintains the melanoma cell population’s ability to proliferate even when blocked by a barrier of CD8+ T cells on the tumor periphery.

Activated CD8+ T cells proliferate (1) into the same chamber they occupy if the chamber is not already full. The rate at which activated CD8+ T cells proliferate is dependent on the total population of CD8+ T cells and on the amount of melanoma cells recently lysed:

$Activated CD8+T cell proliferation rate= k_{pro}\times\left( 1-\frac{N_{Tc}+N_{Te}}{N_{cc}} \right)\times\frac{D_{C}}{D_{\frac{1}{2}}+D_{C}}$

( 4 )

where $k_{pro}$ is the maximum proliferation rate of activated CD8+ T cells; the carrying capacity N_cc_ limits the maximum value of the sum of the populations of the activated CD8+ T cells ($N_{Tc}$ ) and exhausted CD8+ T cells ($N_{Te})$. The last factor incorporates the dependence of the rate on recently lysed melanoma cells (2, 3). We use a Michaelis-Menten functional form: $D_{C}$ denotes the number of melanoma cells killed in the previous 12 hours (effective immune system memory span, $T_{m})$ and $D_{\frac{1}{2}}$ is the Michaelis constant, i.e., when $D_{C}=D_{\frac{1}{2}},$ the rate is halved. The more melanoma cell death, the greater the probability of neoantigens being created invigorating activated CD8+ T cell proliferation and recruitment by way of APCs. This constitutes positive feedback for activated CD8+ T cell population. APCs are not explicitly modeled by our ICS model but their effects on CD8+ T cell proliferation and recruitment are included.

Activated CD8+ T cells are recruited at a rate with the same dependencies on CD8+ T cell carrying capacity and lysed melanoma cells as the activated CD8+ T cell proliferation rate,

$$Activated CD8+T cell recruitment rate=\left( 1-\frac{N_{Tc}+N_{Te}}{N_{cc}} \right)\times\frac{D_{C}}{D_{\frac{1}{2}}+D_{C}}\times r$$

( 5 )

where $r$ is the maximum recruitment rate of activated CD8+ T cells. When recruited, activated CD8+ T cells are randomly placed in a chamber with available space for the cell to occupy. TAMs are recruited at a constant rate, $r_{M}$, and are similarly placed randomly in a lattice chamber with enough space to accommodate the TAM. Exhausted CD8+ T cells and TAMs die at constant rates $\gamma_{E}$ and $\gamma_{M}$ respectively.

In the model, activated CD8+ T cells are transformed into exhausted CD8+ T cells by melanoma cells and TAMs with which they are in contact. In vivo, melanoma cells express PDL1 and PDL2 (4-6) and chronic PD1 activation in CD8+ T cells leads to exhaustion (7). TAMs express PDL1 and PDL2 in the TME (6) and may directly inhibit activated CD8+ T cells (8-11). In CD8+ T cells, PD1 and CTLA4 expression upregulates upon TCR engagement (12, 13). These interactions lead CD8+ T cells to lose cytotoxic and proliferative ability (14).

We account for cell motility in the TMA by allowing CD8+ T cells and TAMs to diffuse on the lattice. A motile cell may move into one of the four nearest neighbor chambers if there is sufficient space for the cell to occupy. The diffusion rate of CD8+ T cells is reduced when in contact with TAMs from $D_{mot}$ to $D_{ad}$. This is a coarse-grained approximation to the myriad of complex processes that promote contact between CD8+ T and melanoma cells. We apply periodic boundary conditions on our lattice which may be viewed as cell migration to and from other regions of the TME not shown in the data. When crossing a lattice boundary, CD8+ T cells have a 50% chance of being removed from the system. We performed simulations to check model prediction of slide response without this random egress of CD8+ T cells at the boundaries and found prediction to be largely unchanged (Table S5).

Sensitivity of patient response to variation of model parameters and ICI therapy

Next, we evaluated the sensitivity of the model predictions against variations of model parameters by perturbing model parameters around their optimized and fixed values (Table 1) independently. We find that the model prediction is most sensitive to variations in the rates at which activated CD8+ T cells are exhausted by melanoma cells and TAMs, melanoma cells proliferate, and activated CD8+ T cells proliferate (Table S1).

We also tested how the TME in a patient would progress if the ICI therapy were not administered using our base ICS model. To effectively assess the effect of withdrawing the ICI therapy, we increased (x1.5) the rate of exhaustion of activated CD8+ T cells, and decreased (x0.666) the rates of lysis of melanoma cells and of proliferation of activated CD8+ T cells from their values in Table 1. CD8+ T cell proliferative and cytotoxic capacities are known to be inhibited by PD-1 (15) and CTLA-4 (16, 17) interactions which also drive CD8+ T cells towards exhaustion (7, 13). The ICI treatments act by blocking the CTLA-4/PD-1 (on CD8+ T cells) interactions with PD-L1 and PD-L2 (on melanoma cells and TAMs) (5, 6). In the absence of anti-PD1/anti-CTLA4 therapy, we reasoned that the exhaustion, cytotoxicity and proliferation rates pertaining to activated CD8+ T cells would vary accordingly from their base values in Table 1. Our simulations showed that melanoma cell dynamics dramatically change when ICI therapy is withdrawn (as described above). Patient slide 06RD who responded well to the ICI therapy and whose response is predicted by the base ICS model becomes a non-responder in simulations without the ICI therapy (Fig. S5f).

Particle Swarm Optimization

A particle swarm optimization algorithm (18) was utilized to search parameter space to minimize the negative log of the prediction success score. The set cognitive, social and inertial parameters were 2, 2 and 0.6 respectively (19). The PSO was done with 10 particles searching through 10 sweeps and $f_{i}(\theta)$ calculated for each parameter set and slide was constructed with 50 simulations.

Cell Segmentation

We segment our cell types following (20). With independent cell locations and marker intensities, we may categorize each cell individually. With the distribution of marker intensities for a given marker across a cell population, we set a threshold above (below) which a cell is considered positive (negative) in that marker. A cell is determined a melanoma cell if it is Sox10+ and S100a+. If a cell is found to be Sox10-, panCK-, and CD31- then we check if it may be a CD8+ T cell. To be an CD8+ T cell, the cell must also be CD3+ and CD8+. We find activated CD8+ T cells by choosing those CD8+ T cells that are PD1+. Finally, an activated CD8+ T cell is found to be exhausted if it is Tim3+ and Lag3+.

Movie S1 R06RD ICS Movie depicts model time-evolution of slide 06RD corresponding to a patient which responded to ICI therapy (separate file). Green dots are melanoma cells, yellow dots are TAMs, cyan dots are activated CD8+ T cells, and red dots are exhausted CD8+ T cells. The system is simulated through about 333 hours from the initial condition.

Movie S2 R16BL ICS Movie depicts model time-evolution of slide 16BL corresponding to a patient which responded to ICI therapy (separate file). Green dots are melanoma cells, yellow dots are TAMs, cyan dots are activated CD8+ T cells, and red dots are exhausted CD8+ T cells. The system is simulated through about 333 hours from the initial condition.

1. A. C. Huang *et al.*, A single dose of neoadjuvant PD-1 blockade predicts clinical outcomes in resectable melanoma. *Nature Medicine* **25**, 454-461 (2019).

2. S. D. Brown *et al.*, Neo-antigens predicted by tumor genome meta-analysis correlate with increased patient survival. *Genome Res* **24**, 743-750 (2014).

3. T. N. Schumacher, R. D. Schreiber, Neoantigens in cancer immunotherapy. *Science* **348**, 69-74 (2015).

4. M. Mandalà, B. Merelli, D. Massi, PD-L1 in melanoma: facts and myths. *Melanoma Manag* **3**, 187-194 (2016).

5. H. Dong *et al.*, Tumor-associated B7-H1 promotes T-cell apoptosis: A potential mechanism of immune evasion. *Nature Medicine* **8**, 793-800 (2002).

6. J. M. Obeid *et al.*, PD-L1, PD-L2 and PD-1 expression in metastatic melanoma: Correlation with tumor-infiltrating immune cells and clinical outcome. *Oncoimmunology* **5**, e1235107 (2016).

7. B. Bengsch *et al.*, Bioenergetic Insufficiencies Due to Metabolic Alterations Regulated by the Inhibitory Receptor PD-1 Are an Early Driver of CD8+ T Cell Exhaustion. *Immunity* **45**, 358-373 (2016).

8. K. Kersten *et al.*, Spatiotemporal co-dependency between macrophages and exhausted CD8+ T cells in cancer. *Cancer Cell* **40**, 624-638.e629 (2022).

9. I. Kryczek  *et al.*, B7-H4 expression identifies a novel suppressive macrophage population in human ovarian carcinoma. *Journal of Experimental Medicine* **203**, 871-881 (2006).

10. W. Zou, J. D. Wolchok, L. Chen, PD-L1 (B7-H1) and PD-1 pathway blockade for cancer therapy: Mechanisms, response biomarkers, and combinations. *Science Translational Medicine* **8**, 328rv324-328rv324 (2016).

11. B. Boldajipour, A. Nelson, M. F. Krummel, Tumor-infiltrating lymphocytes are dynamically desensitized to antigen but are maintained by homeostatic cytokine. *JCI Insight* **1** (2016).

12. Y. Agata *et al.*, Expression of the PD-1 antigen on the surface of stimulated mouse T and B lymphocytes. *International Immunology* **8**, 765-772 (1996).

13. J. G. Egen, J. P. Allison, Cytotoxic T Lymphocyte Antigen-4 Accumulation in the Immunological Synapse Is Regulated by TCR Signal Strength. *Immunity* **16**, 23-35 (2002).

14. C. U. Blank *et al.*, Defining ‘T cell exhaustion’. *Nature Reviews Immunology* **19**, 665-674 (2019).

15. M. J. Butte, M. E. Keir, T. B. Phamduy, A. H. Sharpe, G. J. Freeman, Programmed death-1 ligand 1 interacts specifically with the B7-1 costimulatory molecule to inhibit T cell responses. *Immunity* **27**, 111-122 (2007).

16. E. Hui *et al.*, T cell costimulatory receptor CD28 is a primary target for PD-1–mediated inhibition. *Science* **355**, 1428-1433 (2017).

17. S. C. Wei, C. R. Duffy, J. P. Allison, Fundamental Mechanisms of Immune Checkpoint Blockade Therapy. *Cancer Discovery* **8**, 1069-1086 (2018).

18. J. Kennedy, "Swarm intelligence" in Handbook of nature-inspired and innovative computing: integrating classical models with emerging technologies. (Springer, 2006), pp. 187-219.

19. S. E. Selvan *et al.*, Parameter Estimation in Stochastic Mammogram Model by Heuristic Optimization Techniques. *IEEE Transactions on Information Technology in Biomedicine* **10**, 685-695 (2006).

20. A. J. Nirmal *et al.*, The Spatial Landscape of Progression and Immunoediting in Primary Melanoma at Single-Cell Resolution. *Cancer Discovery* **12**, 1518-1541 (2022).

21. D. A. Rew, G. D. Wilson, Cell production rates in human tissues and tumours and their significance. Part II: clinical data. *European Journal of Surgical Oncology (EJSO)* **26**, 405-417 (2000).

22. C. Gong *et al.*, A computational multiscale agent-based model for simulating spatio-temporal tumour immune response to PD1 and PDL1 inhibition. *J R Soc Interface* **14** (2017).

23. K. E. Yost, H. Y. Chang, A. T. Satpathy, Recruiting T cells in cancer immunotherapy. *Science* **372**, 130-131 (2021).

24. M. H. Spitzer *et al.*, Systemic Immunity Is Required for Effective Cancer Immunotherapy. *Cell* **168**, 487-502.e415 (2017).

25. I. Mellman, D. S. Chen, T. Powles, S. J. Turley, The cancer-immunity cycle: Indication, genotype, and immunotype. *Immunity* **56**, 2188-2205 (2023).

26. C. Kurts, H. Kosaka, F. R. Carbone, J. F. A. P. Miller, W. R. Heath, Class I–restricted Cross-Presentation of Exogenous Self-Antigens Leads to Deletion of Autoreactive CD8+ T Cells. *Journal of Experimental Medicine* **186**, 239-245 (1997).

27. H. Yoon, T. S. Kim, T. J. Braciale, The Cell Cycle Time of CD8+ T Cells Responding In Vivo Is Controlled by the Type of Antigenic Stimulus. *PLOS ONE* **5**, e15423 (2010).

28. L. C. Eisenlohr, L. Huang, T. N. Golovina, Rethinking peptide supply to MHC class I molecules. *Nature Reviews Immunology* **7**, 403-410 (2007).

29. B. Weigelin *et al.*, Cytotoxic T cells are able to efficiently eliminate cancer cells by additive cytotoxicity. *Nature Communications* **12**, 5217 (2021).

30. J. N. Kather *et al.*, In Silico Modeling of Immunotherapy and Stroma-Targeting Therapies in Human Colorectal Cancer. *Cancer Research* **77**, 6442-6452 (2017).

31. H. Raskov, A. Orhan, J. P. Christensen, I. Gögenur, Cytotoxic CD8+ T cells in cancer and cancer immunotherapy. *British Journal of Cancer* **124**, 359-367 (2021).

32. M. L. Dustin, The Immunological Synapse. *Cancer Immunology Research* **2**, 1023-1033 (2014).

33. R. J. De Boer, D. Homann, A. S. Perelson, Different Dynamics of CD4+ and CD8+ T Cell Responses During and After Acute Lymphocytic Choriomeningitis Virus Infection 1. *The Journal of Immunology* **171**, 3928-3935 (2003).

34. C. G. Cess, S. D. Finley, Multi-scale modeling of macrophage—T cell interactions within the tumor microenvironment. *PLOS Computational Biology* **16**, e1008519 (2020).

35. T. Hourani *et al.*, Tumor Associated Macrophages: Origin, Recruitment, Phenotypic Diversity, and Targeting. *Front Oncol* **11**, 788365 (2021).

36. J. Cosgrove, L. S. P. Hustin, R. J. de Boer, L. Perié, Hematopoiesis in numbers. *Trends Immunol* **42**, 1100-1112 (2021).

37. J. Lahoz-Beneytez *et al.*, Human neutrophil kinetics: modeling of stable isotope labeling data supports short blood neutrophil half-lives. *Blood* **127**, 3431-3438 (2016).

38. C. Zhao *et al.*, DNA methyltransferase 1 deficiency improves macrophage motility and wound healing by ameliorating cholesterol accumulation. *npj Regenerative Medicine* **8**, 29 (2023).

39. H. Yano *et al.*, Reduction of Real-Time Imaging of M1 Macrophage Chemotaxis toward Damaged Muscle Cells is PI3K-Dependent. *Antioxidants (Basel)* **7** (2018).

40. E. Au - van den Bos, S. Au - Walbaum, M. Au - Horsthemke, A. C. Au - Bachg, P. J. Au - Hanley, Time-lapse Imaging of Mouse Macrophage Chemotaxis. *JoVE* doi:10.3791/60750, e60750 (2020).
