# Supplementary material for: Spatial organization and stochastic fluctuations of immune cells impact clinical responsiveness to immunotherapy in melanoma patients
Source: PNAS Nexus. 2024 Nov 26;3(12):pgae539. doi: 10.1093/pnasnexus/pgae539 (PMC11642613; doi:10.1093/pnasnexus/pgae539)
Supplement: pgae539_Supplementary_Data [file pgae539_supplementary_data.zip › PNAS_Nexus_Figure_S2_Updated.pdf]

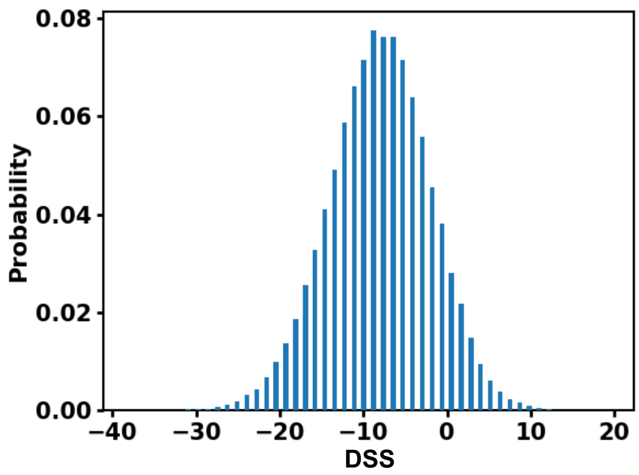

Figure S2. Melanoma cell exhaustion of activated CD8+ T cell hypothesis test points to relevance of activated CD8+ T cell exhaustion by melanoma cells. DSS distribution from 100,000 bootstraps for the hypothesis "prediction power of the full model is the same as in the model without melanoma exhaustion of activated CD8+ T cells". Hypothesis is rejected with p-value 0.088.
